# Cortical morphology changes in default mode network regions as predictors of cognitive decline in relation to amyloid and tau deposits

**DOI:** 10.1093/braincomms/fcaf320

**Published:** 2025-08-28

**Authors:** Arianna Menardi, Ceren Saglam, Beatrice La Rocca, Diego Cecchin, Annalena Venneri, Annachiara Cagnin, Antonino Vallesi

**Affiliations:** Department of Neuroscience, University of Padova, 35128 Padova, Italy; Padova Neuroscience Center, University of Padova, 35131 Padova, Italy; Department of General Psychology, University of Padova, 35131 Padova, Italy; Department of General Psychology, University of Padova, 35131 Padova, Italy; Padova Neuroscience Center, University of Padova, 35131 Padova, Italy; Department of Medicine (DIMED), University Hospital of Padova, 35128 Padova, Italy; Department of Medicine and Surgery, University of Parma, 43125 Parma, Italy; Department of Neuroscience, University of Sheffield, S10 2TN Sheffield, UK; Department of Neuroscience, University of Padova, 35128 Padova, Italy; Padova Neuroscience Center, University of Padova, 35131 Padova, Italy; Department of Neuroscience, University of Padova, 35128 Padova, Italy; Padova Neuroscience Center, University of Padova, 35131 Padova, Italy

**Keywords:** Default Mode Network, structural morphology, episodic memory, Alzheimer’s disease

## Abstract

Alzheimer’s disease can be classified based on amyloid, tau and neurodegeneration status. The Default Mode Network is notably vulnerable to these processes, making early structural alterations in this network of particular interest for identifying prodromal biomarkers. In this longitudinal cross-sectional study, we analysed data from 279 participants in the Alzheimer’s Disease Neuroimaging Initiative (mean age = 73.7 ± 9 years, 53.2% males). Structural measures—sulcal depth, gyrification and cortical thickness—were extracted for all Default Mode Network regions. Their ability to predict memory performance (encoding, retrieval and recall) was tested at baseline and 2-year follow-up by means of multiple linear regression models, which were all corrected for the risk of multiple comparisons. Covariates included Mini Mental State Examination scores, amyloid status and regional tau burden, to examine interactions with structural changes. Our results showed distinct Default Mode Network alteration patterns based on tau burden and amyloid status, highlighting patterns of morphological features with different susceptibility to proteinopathy. In individuals with concordant (both positive or both negative) amyloid and tau status, preserved structural integrity and complexity were linked to better cognitive performance and appeared protective against decline. However, mainly negative associations were instead observed in individuals with discordant amyloid or tau status (i.e. positive for only either amyloid or tau accumulation). We discuss these findings as a possible reflection of a mismatch between abnormal protein accumulation and structural damage in these populations. The multimodal nature of this study helps clarifying the heterogeneous findings reported in existing literature regarding structural integrity and cognitive outcomes in Alzheimer’s disease.

## Teaser

Menardi *et al.* used multimodal imaging data to model cognitive decline combining neuroanatomical, PET-derived biomarkers and subject-specific variables. While preserved integrity and complexity appeared protective against decline, some regions showed unexpected, inverted patterns. These may reflect mismatches between tau buildup and structural damage or compensatory brain folding mechanisms countering neurodegeneration.

## Graphical Abstract

**Graphical Abstract:**
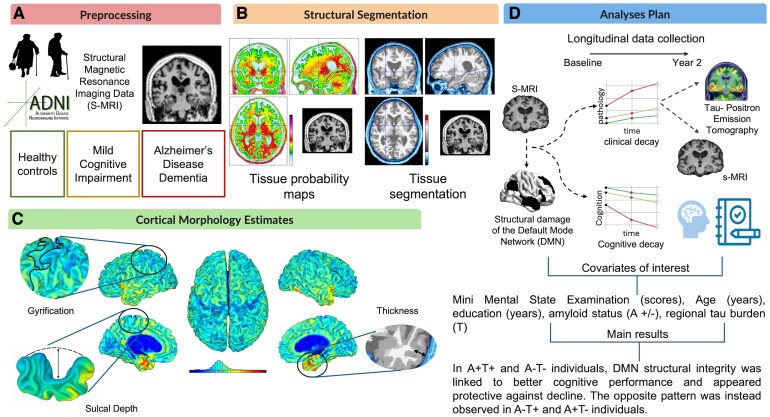

## Introduction

Alzheimer’s disease is a leading cause of dementia in the elderly,^1^ characterized by progressive neurodegeneration driven by amyloid beta plaques and neurofibrillary tau tangles.^2,3^ To capture this complexity, the ATN model was introduced,^4^ categorizing biomarkers into amyloid pathology (A), phosphorylated-tau (T), and neurodegeneration (N), with individuals classified as positive or negative for each.^5^ All three biomarkers appear in significant overlap with regions of the Default Mode Network (DMN),^2,6-8^ a resting-state network showing reduced activation during goal-directed tasks but increased activity at rest.^9,10^ Since the DMN is highly metabolically demanding^11^ and because amyloid tends to accumulate in metabolically active regions,^12,13^ early functional disruption of this network can be observed even before clinical symptoms.^6,14^ Recent work has also linked tau deposition to DMN disruption,^15-18^ with tau pathology correlating more closely with cognitive decline and network breakdowns than amyloid.^16,19^ However, while DMN functional and metabolic disruptions are well documented, structural changes remain less understood. Surface-Based Morphometry (SBM) provides valuable tools for exploring the relationship between grey matter (GM) surface morphology and stages of Alzheimer’s disease.^20-22^ Indeed, beyond measuring cortical atrophy, commonly used SBM measures, such as cortical thickness, gyrification, and sulcal depth, enable the investigation of more fine-grained patterns of alteration within the cortical folding.^23^ However, inconsistent findings have been reported in the literature regarding the sensitivity of these measures and the directionality of their changes in respect to behaviour.

Cortical thickness, the distance between the pial surface and white matter (WM) boundary,^24^ is significantly reduced in patients with Alzheimer’s disease compared with controls, especially in temporal, parietal, and limbic areas.^23,25,26^ Thinning correlates with symptom severity from early stages^25^ and in Mild Cognitive Impairment (MCI), future Alzheimer’s disease converters show pronounced cortical thinning in Alzheimer’s disease–related regions.^23^ However, longitudinal evidence suggests that progressive thinning of the temporal cortices is linked to memory impairments in amnestic MCI patients, but not in Alzheimer’s disease–dementia, for whom the association with cognitive decline is better captured by other SBM measures.^23^ Similarly, in the healthy elderly population, cortical thickness measures have been reported as a weaker predictor of future memory decline, compared with tau burden.^27^ This argues in favour of more sensitive measures of cortical folding alterations, such as gyrification and sulcal depth.

Gyrification, reflecting the degree of cortical folding,^28^ underpins the brain's computational efficiency.^29^ In healthy adults, gyrification relates to brain volume and higher-order cognitive functions, particularly in frontal cortices.^29^ Findings on the Alzheimer’s disease continuum are mixed: mostly, these report reduced gyrification in Alzheimer’s disease compared with MCI and controls, particularly in posterior-medial temporal regions.^26,30^ However, more recent studies have reported both higher and lower gyrification—for example, lower gyrification in the bilateral insular cortex and higher in the entorhinal cortex in Alzheimer’s disease–dementia patients compared with healthy individuals.^31^ Higher insular gyrification was associated with better memory in Alzheimer’s disease–dementia but not in MCI or healthy groups.^31^ In MCI, higher gyrification in the right inferior frontal cortex (IFC), compared with cognitively normal individuals, has instead been linked to poorer memory performance.^32^ Coleman *et al*.^33^ also found both negative and positive associations between gyrification and behaviour—verbal learning and language were negatively correlated with gyrification in the temporal regions, while executive function showed a positive association.

Finally, sulcal depth measures the distance between the central surface of the cortex and the hemispheric hull,^23,33^ offering insights into atrophy and folding. Sulcal widening increases with age^34^ and it is associated with cognitive decline,^30,35,36^ with greater widening and reduced sulcal depth observed in MCI and Alzheimer’s disease–dementia patients, particularly in temporal regions.^23,30,37,38^ Some studies suggest temporoparietal sulcal width may be more sensitive than traditional Alzheimer’s disease markers like hippocampal volume,^35,39^ cortical thickness and gyral WM.^37,40^ However, limited studies exist, and further research is needed to connect sulcal measures with behaviour.

In the present study, we tried to bridge the gap and overcome some limitations and missing knowledge in the literature on the association between structural alterations in Alzheimer’s disease and memory performance. Indeed, extensive evidence supports the notion that episodic memory does not only rely on the activity of single regions (i.e. medial temporal), but it involves a more distributed network of structures, particularly involving the DMN.^10,41-45^ We conducted our investigation taking the ATN model into account, hypothesizing that a more disrupted DMN due to the presence (and extent) of amyloid and tau proteinopathies might present a weaker association with behaviour, compared with milder Alzheimer’s disease stages or healthy aging. This might explain the prior mixed findings on the relationship between thickness, gyrification, sulcal depth, and behaviour that we specifically tested here with reference to baseline and future memory decline, looking at a 2-year follow-up period. We also decided to test the association between baseline cortical morphology properties and future regional tau build-ups. This was based on the rationale that if tau pathology spreads to regions based on the strength of their functional connection^46^ and if functional connectivity is influenced by the underlying structurally integrity,^47-49^ then we could expect greater future tau accumulation in regions more structurally intact at baseline, rather than in regions already undergoing atrophy. At the same time, the brain reserve hypothesis suggests that greater availability of a given substrate (i.e. greater GM volume, cortical thickness, etc.) might act as a neural protective factor,^50^ which would imply the opposite relationship between those two variables.

Finally, to determine the specificity of the DMN findings, we compared them with those of a neighbouring network, the frontoparietal network (FPN). We believe that the multimodal, longitudinal nature of our study, with in-depth neuroimaging and memory assessments adds value to prior studies in the literature.

## Materials and methods

### Participants

Our initial sample consisted of 575 participants from the third cohort of the Alzheimer Disease Neuroimaging Initiative (ADNI, https://adni.loni.usc.edu/) (HC = 183, MCI = 321, Alzheimer’s disease = 71) for which structural, functional and cognitive variables were available for at least two timepoints (baseline and 2-year follow-up). Data used in the preparation of this article were obtained from the ADNI database (adni.loni.usc.edu). The ADNI was launched in 2003 as a public–private partnership, led by Principal Investigator Michael W. Weiner, MD. The primary goal of ADNI has been to test whether serial magnetic resonance imaging (MRI), positron emission tomography (PET), other biological markers, and clinical and neuropsychological assessment can be combined to measure the progression of MCI and early Alzheimer’s disease. Data collection procedures were approved by the institutional review boards of the participating centers to the ADNI project (https://adni.loni.usc.edu/help-faqs/adni-documentation/). No IRB approval was needed for the current study. All participants recruited by the ADNI consortium gave their informed consent prior to participating to the data collection in accordance with the Declaration of Helsinki (https://adni.loni.usc.edu/help-faqs/adni-documentation/).

Due to concerns on the impact of motion on the data, participants with a framewise displacement (FD) greater than 0.5 mm were disregarded from further analysis (*n* = 13 MCI; *n* = 5 Alzheimer’s disease-dementia). FD was computed from functional MRI data acquired in the same session. FD was then used as a proxy to exclude individuals with possible high motion in structural scanners as well, since prior investigations have linked greater movement to reduced morphological estimates.^51^ Our participant group size was reduced further to retain only participants with available amyloid and tau PET data acquired within 6 months from the MRI acquisition (baseline: *n* = 129 A+, *n* = 150 A−; Y2: *n* = 78 A+, *n* = 98 A−). Demographic data of the final sample are summarized in Table 1.

**Table 1:** Participants’ demographics

	Age (M ± SD)	Education (M ± SD)	Sex (% Males)	MMSE (M ± SD)	FD (mm) (M ± SD)
**HC**					
A− (*n* = 73)	71.7 ± 6.7	16.73 ± 2.3	47%	29.2 ± 0.9	0.14 ± 0.09
A+ (*n* = 35)	73.2 ± 7.7	16.2 ± 1.9	37%	28.7 ± 1.7	0.14 ± 0.07
**MCI**					
A− (*n* = 77)	72.5 ± 8.9	16.19 ± 2.8	66%	28.3 ± 1.7	0.19 ± 0.13
A+ (*n* = 73)	75.6 ± 7.7	16.3 ± 2.3	49%	26.3 ± 3.7	0.18 ± 0.12
**Alzheimer’s disease–dementia**					
A+ (*n* = 21)	75.4 ± 13.9	16.7 ± 4.16	67%	23.7 ± 1.1	0.23 ± 0.18

A+ refers to amyloid status positivity, whereas A– refers to the lack of such biomarker.

### Cognitive measures

In the present study, we relied on Mini Mental State Examination (MMSE) scores to define the clinical severity of our participants along a continuum, thus without stratifying them based on specific diagnostic labels. As per the assessment of their memory function, we relied on a recently introduced measure derived from Bayesian modelling, informative of participants’ performance across all stages of memory formation and consolidation: encoding, retrieval and recall. A brief description of such measures is provided next.

#### Mini Mental State Examination

The MMSE is a widely used paper-based screening tool that aims to grade the cognitive state of an individual.^52,53^ It assesses multiple cognitive domains, including orientation, memory, attention, calculation, recall, and language. The total score ranges from 0 to 30, with higher scores indicating better cognitive function.^52^ Suggested cut-off scores for classifying cognitive status are as follows: no cognitive impairment (24–30), MCI (18–23) and severe cognitive impairment (0–17).^54^

#### Embic’s digital cognitive biomarkers

In line with neuroimaging and biomarkers-detection advancement, there is also the need to develop more sensitive neuropsychological markers.^55^ In this respect, the Embic’s Digital Cognitive Biomarkers (EMBIC-DCB) present quantitative scores that aim at providing more sensitive indexes of verbal memory performance, capable of detecting even subtle alterations.^55^ These probabilities are derived from the use of a specific Hidden Markov Model, known as Hierarchical Bayesian model, based on participants’ performance on various wordlist memory tests. Successful or erroneous patterns in performance are used by the model to estimate the probability of correct encoding and/or retrieval from a subset of latent storage processes, resulting in probability estimates of latent memory functions: encoding (*N*), retrieval (R) and recall (M).

From their combined estimates, the model generates seven DCBs in total. Four measures are used to represent different encoding stages: ‘N1—Probability of encoding into the durably learned state’, ‘N2—Probability of encoding into the transiently state’, ‘N3—Probability of encoding into the durably learned state, following previous transient learning (N2)’, ‘N4—Probability of encoding into the durably learned state, due to successful retrieval (R1) from transiently learned state’. There are then three measures for retrieval: ‘R1—Probability of retrieving from the transiently learned state’, ‘R2—Probability of retrieving from the durably learned state’, ‘R3-Probability of retrieving from the durably learned state after a 5-minute delay with distraction’. Finally, there are three measures for recall: ‘M1—Probability of immediate recall of a non-durably stored episodic memory’, ‘M2—Probability of immediate recall of a durably stored episodic memory’, ‘M3—Probability of delayed recall of a durably stored episodic memory’. More details are available in Bruno *et al*.^55^ and in the ADNI Documents’s repository (https://adni.loni.usc.edu/).

Given the recent introduction of such measures, very limited literature is available. However, it has been reported so far that EMBIC-DCB scores outperform other recall measures and strongly predict clinical dementia scores at a 36-month follow-up.^55^ Furthermore, EMBIC-DCB have been shown to be sensitive in predicting HC to MCI and MCI to Alzheimer’s disease–dementia conversions, as well as to be related to volumetric indexes of atrophy in the hippocampus and entorhinal cortices, and to show steeper decline values in the presence of positive CSF markers of pathology.^56^ To our knowledge, this would be the third study assessing the validity of such neuropsychological measures in the Alzheimer’s disease continuum and the first one to put it in relation with a variety of cortical morphology measures.

### Structural MRI

T1w Accelerate Sagittal MPRAGE images were acquired on a 3T Siemens PRISMA scanner (TR = 2300 ms, TE = 2.95 ms, slice thickness = 1.2 mm, Flip Angle = 9°, 176 × 240 × 256 matrix, voxel size = 1.05 × 1.05 × 1.20 mm^3^). More information on acquisition procedures are available in ADNI (https://adni.loni.usc.edu/help-faqs/adni-documentation/). Regional structural measures of cortical thickness, gyrification and sulcal depth were extracted using CAT12 (http://www.neuro.uni-jena.de/cat),^57^ implemented in SPM12 (https://www.fil.ion.ucl.ac.uk/spm/software/spm12/), which was run in MATLAB 2023a (MathWorks, Natick, MA).

Steps included skull stripping and segmentation of T1 images into different tissue classes (GM; WM; CSF). Surface mesh reconstruction is handled based on a projection-based thickness method, which relies on the GM/WM boundary and pial surface estimation.^24^ Topological defects are then corrected by means of spherical harmonics, a method that involves a two-step process of first relaying on original MRI intensity to select either a ‘fill’ or ‘cut’ operation for each topological defect, followed by the application of a low-pass filtered alternative reconstruction on the areas containing defects.^58^ Spherical mapping is then applied to the reconstructed cortical surface, which is mapped onto a standardized spherical representation to allow inter-subject analysis.^59^ As per SBM measures, a projection-based method is used to derive cortical thickness estimates while handling partial volume effects, sulcal blurring and sulcal asymmetries.^24^ Gyrification indexes are extracted based on the absolute mean curvature,^60^ while sulcal depth measurements are estimated based on the distance between the central surface of the cortex and the hemispheric hull.

Maps were resampled to a high-resolution 32k mesh and then smoothed by applying a 15 mm and a 20 mm full width at half maximum Gaussian Kernel to thickness and gyrification maps respectively, following CAT software recommendations. While smoothing is applied to increase the signal to noise ratio, it might hinder the spatial specificity of the measures we extract. While this might be a concern of which to have awareness, large filters are needed for cortical folding estimates as their size has to exceed the distance between the gyral crown and the sulcus fundus (see CAT12 manual). All thickness, gyrification and sulcal depth indexes were extracted at the single parcel level based on the Schaefer 400 surface atlas,^61^ which was used to identify the brain parcels associated with the DMN on the individual cortex. Weighted Image Quality Rating scores made available by CAT12 suggested good image quality for all participants in our sample (see section 1 in the Supplementary Materials and Supplementary Fig. 1).

Furthermore, association and dissociation between all metrics considered in this study, as well as a comparison with more ‘traditional’ GM volume estimates, are available in the Supplementary Materials (section 2).

### PET imaging

For the purpose of this study, we retrieved the Amyloid (A) +/− categorization made available by ADNI (ADNI_UCBerkeley_AmyloidPET), which is computed as the overall standard uptake value ratios (SUVRs) burden following PET intensity normalization by the whole cerebellum. Of note, amyloid PET data were acquired with two different tracers: florbetaben (FBB) and florbetapir (FBP). The ADNI_UCBerkeley_AmyloidPET document makes available dichotomization for all patients (positive and negative amyloid status) based on a cut-off of 1.11SUVR for FBP and 1.08SUVR for FBB tracers.

As for the estimate of tau deposits, we ensured that all participants underwent tau PET (ADNI_UCBerkeley_TauPET) with the same tracer (flortaucipir) and that data were corrected for partial volume effects. Tau data were also intensity-normalized using the inferior cerebellar GM as reference. Since tau SUVR data are provided based on the Desikan–Killiany Atlas,^62^ we performed a vertex-to-vertex correspondence analysis between the Schaefer and the Desikan–Killiany atlases at the surface level to assign tau deposits to the Schaefer parcels based on their overlap with the Desikan–Killiany parcels. This was computed as the weighted average of tau PET SUVR based on the number of overlapping vertices. For both amyloid and tau data, we considered only participants who had a PET scan within 6 months of acquisition from the MRI data.

### Statistical analysis

Statistical analyses were run in MATLAB 2023a (The Mathworks, Inc., Natick, MA, United States). Given the impact of amyloid positivity in neurodegenerative patterns, all statistical analyses were carried out separately in A+ and A– individuals. In the latter group, we only considered individuals with MMSE > 26 (seven participants were excluded with MMSE < 26) to provide a sort of normative group with no markers of Alzheimer’s disease pathology and overall intact cognition.

One-way Analysis of Variance (ANOVA) models were first run to control for the presence of significant difference among HC, MCI and Alzheimer’s disease–dementia participants in the variables of education, age and MMSE in A+ individuals. On the other hand, a two-sample *t*-test was used to assess the presence of significant differences in A– participants (HC A– versus MCI A−). Multiple linear regression analyses were then used to test the power of structural measures (gyrification, thickness, sulcal depth) for predicting EMBIC-DCB performance at both baseline and follow-up. In order to decrease the number of statistical comparisons, encoding (N1, N2, N3, N4), retrieval (R1, R2, R3) and recall (M1, M2, M3) scores from the original EMBIC-DCB scale were averaged together into single measures (N, R, M), in line with recent published evidence.^55^ To control for the influence of additional factors, the variables age, MMSE, education and regional tau deposits were inserted as continuous covariates of interest in the analyses, together with their interaction with the predictors. Variance inflation factor (VIF) measures were always inspected to ensure no risk of multicollinearity in the models (VIF∼1). Furthermore, outliers were removed based on the models’ residuals, as determined by a cut-off of ± 3 scaled absolute deviations from the median (average *n* = 3 participants). As a result, separate models were run for each memory measure of interest (N, R, M) and each DMN parcel (*n* = 91) at both baseline and follow-up (Year 2—Y2). To control for the risk of multiple comparisons, only models surviving FDR correction (*p* = 0.05) were considered. All models report the associated $R^{2}$ values to help address the effect sizes reported. The same analyses were run on FPN nodes (see section 4 in the Supplementary Materials, Supplementary Tables 4–6 and Supplementary Figs 3–5).

**Figure 1:**
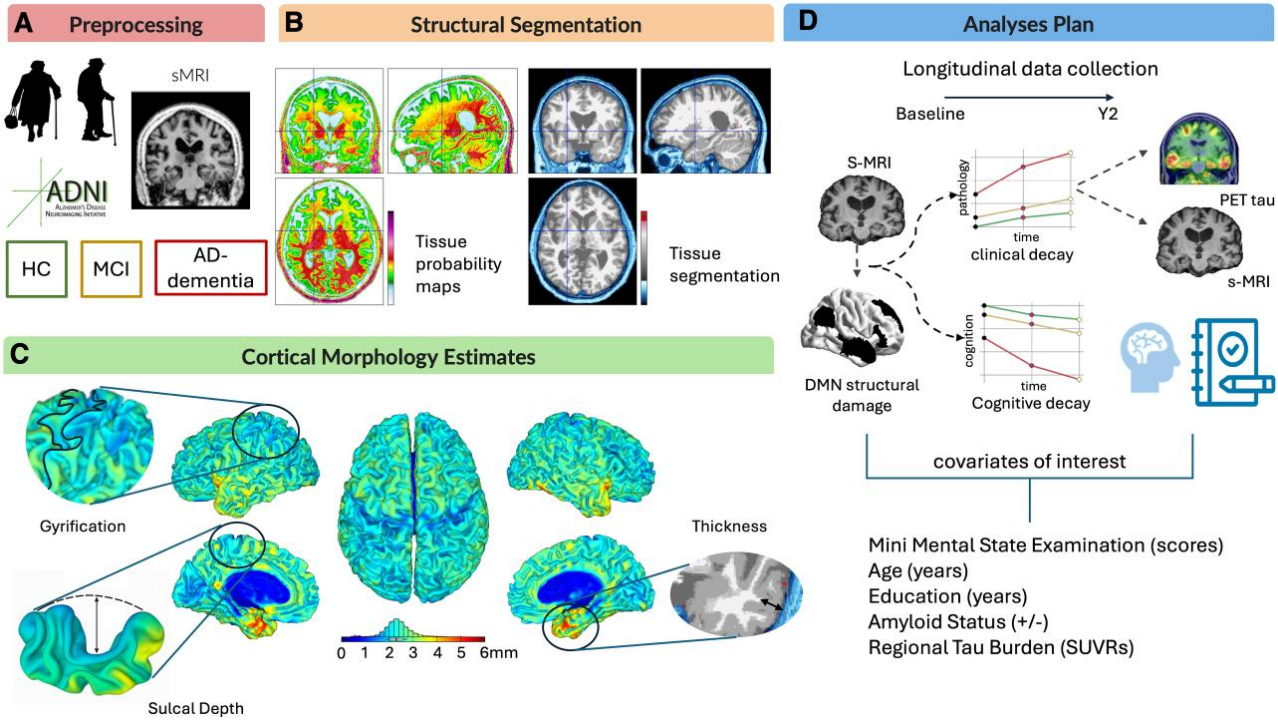
**Methodological workflow.** Structural MRI data were retrieved from the ADNI database (**A**). All images were preprocessed and segmented into their main tissue components (grey matter, white matter and cerebrospinal fluid) (**B**) in order to derive cortical morphological measures of thickness, gyrification and sulcal depth (**C**). Morphological measures of the DMN were used in our analyses to try to predict both clinical worsening and cognitive decay in consideration of several individual-specific variables (MMSE scores, age, education, amyloid status and regional tau burden (**D**). AD, Alzheimer’s Disease; ADNI, Alzheimer’s Disease Neuroimaging Initiative; DMN, default mode network; HC, healthy controls; MCI, mild cognitive impairment; MMSE, Mini Mental State Examination; s-MRI, structural magnetic resonance imaging; PET, positron emission tomography; Y2, year 2 follow-up.

Finally, we conducted Pearson’s correlation analyses to assess the association between structural alterations at baseline and tau deposits at the 2-year follow-up. An overall representation of the methodological workflow is presented in Fig. 1.

## Results

### Relationship between baseline structural alterations and memory performance

#### Amyloid positive status

**Figure 2:**
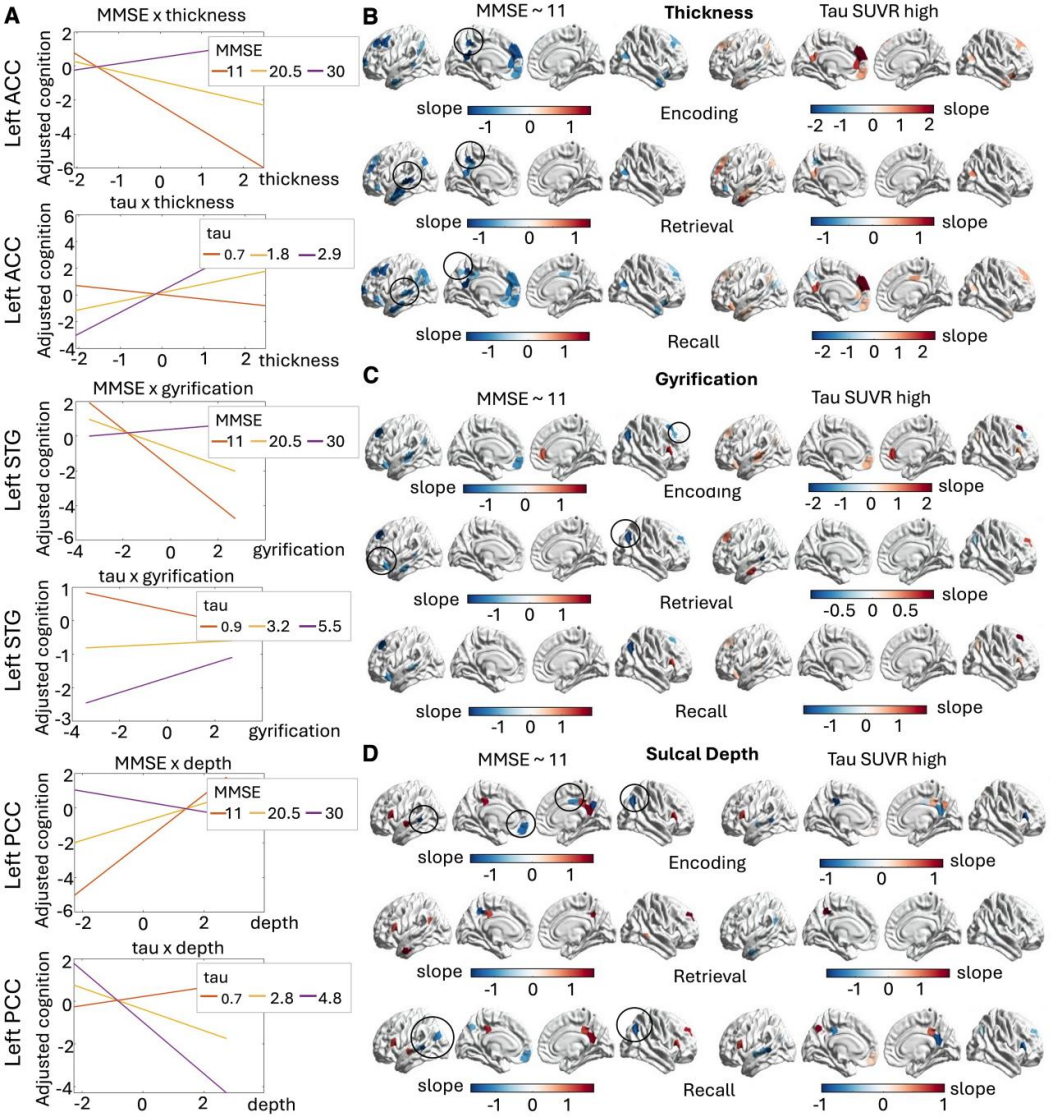
**Cortical morphology measures in relation to memory performance in A+ individuals.** Plot of the results of the multiple linear regression models (*n* = 129 individuals). Interaction plots depicting the exemplificative patterns of cortical regions are shown in reference to MMSE and regional tau deposits (**A**). The slope values for all nodes with significant interaction with MMSE scores for thickness (**B**), gyrification (**C**) and sulcal depth (**D**) are shown on the brain surface. Slope values of the interaction between thickness, gyrification, sulcal depth and tau SUVRs in predicting memory performance are also shown. Regions marked with a circle indicate DMN parcels with opposite interaction effects, unexplained by the degree of regional tau deposits. ACC, anterior cingulate cortex; MMSE, mini mental state examination; PCC, posterior cingulate cortex; STG, superior temporal gyrus; SUVR, PET standard uptake values ratios.

All structural measures emerged as being significantly associated with baseline encoding, retrieval and recall performance, with particularly marked patterns in the most severe patients (see Fig. 2).

For instance, cortical thickness and gyrification values of several DMN regions were significantly associated with memory performance as a function of MMSE scores. In individuals with an A+ status but preserved cognitive functioning (i.e. high MMSE scores) we observed a weak positive relationship, suggesting that higher thickness and gyrification favourably sustain memory performance (see interaction plots in Fig. 2 A). In individuals with the lowest MMSE scores, the relationship between thickness/gyrification and memory performance was better captured when simultaneously considering regional tau deposits. In more detail, in regions with particularly high levels of tau, residual cortical thickness and gyrification were positively associated with higher encoding, retrieval and recall scores (see Fig. 2 B and C). This suggests that even in the face of high tau burden, residual thickness and gyrification still correlate with better memory performance. With respect to sulcal depth measures, positive associations were observed for the bilateral pars opercularis (PO), left middle temporal gyrus (MTG) and superior temporal gyrus (STG) and bilateral PCC in individuals with low MMSE scores and these predicted encoding, retrieval and recall performance (see Fig. 2 D). As sulcal depth has been observed to become shallower with increased pathological burden,^37^ higher sulcal depth in relation to better performance might be indicative of more preserved complexity in the cortical folding patterns.

Of note, for all morphological measures we also observed a selective number of regions for which the patterns appeared inverted and not explained by the regional tau burden (marked by circles in Fig. 2). For instance, lower thickness values of the left STG and left precuneus appeared to be associated with higher retrieval and recall scores. Similar trends were observed for the gyrification of the left IFC and of small regions within the right dorsolateral prefrontal cortex (DLPFC) and right IPL, again suggesting that lower gyrification of such regions was associated with better baseline memory performance. Finally, only the sulcal depth of the left ACC/IFC, bilateral IPL and right PCC was observed to be negatively associated with memory performance, opposite to the positive association seen for other DMN regions.

In summary, we observed that several DMN regions are significantly associated with baseline encoding, retrieval and recall performance. In cognitively intact individuals (with high MMSE scores), greater thickness and gyrification—and lower sulcal depth—are associated with better memory performance. On the other hand, this relationship is reversed (i.e. negative) in more severe participants (i.e. those with low MMSE scores). However, when considering the interaction between morphology measures and regional tau burden, we observed that residual integrity of the cortex was also positively linked with better memory performance in this more severe group. Of note, we observed a similar pattern even when the analyses were conducted on the FPN nodes (see Supplementary Materials, section 4). However, only FPN thickness was a significant predictor of encoding and recall performance, with little contribution from gyrification and sulcal depth measures. All models’ statistics are available in Supplementary Table 1 in the Supplementary Materials.

#### Amyloid negative status

Although we only selected A– participants who presented a MMSE > 26, we observed high variability in their levels of tau deposits within DMN regions (range = 0.9–1.61 SUVR). As such, here we investigated the predictive power of all regions presenting significant interaction between their structural indexes and tau deposits with respect to memory performance.

**Figure 3:**
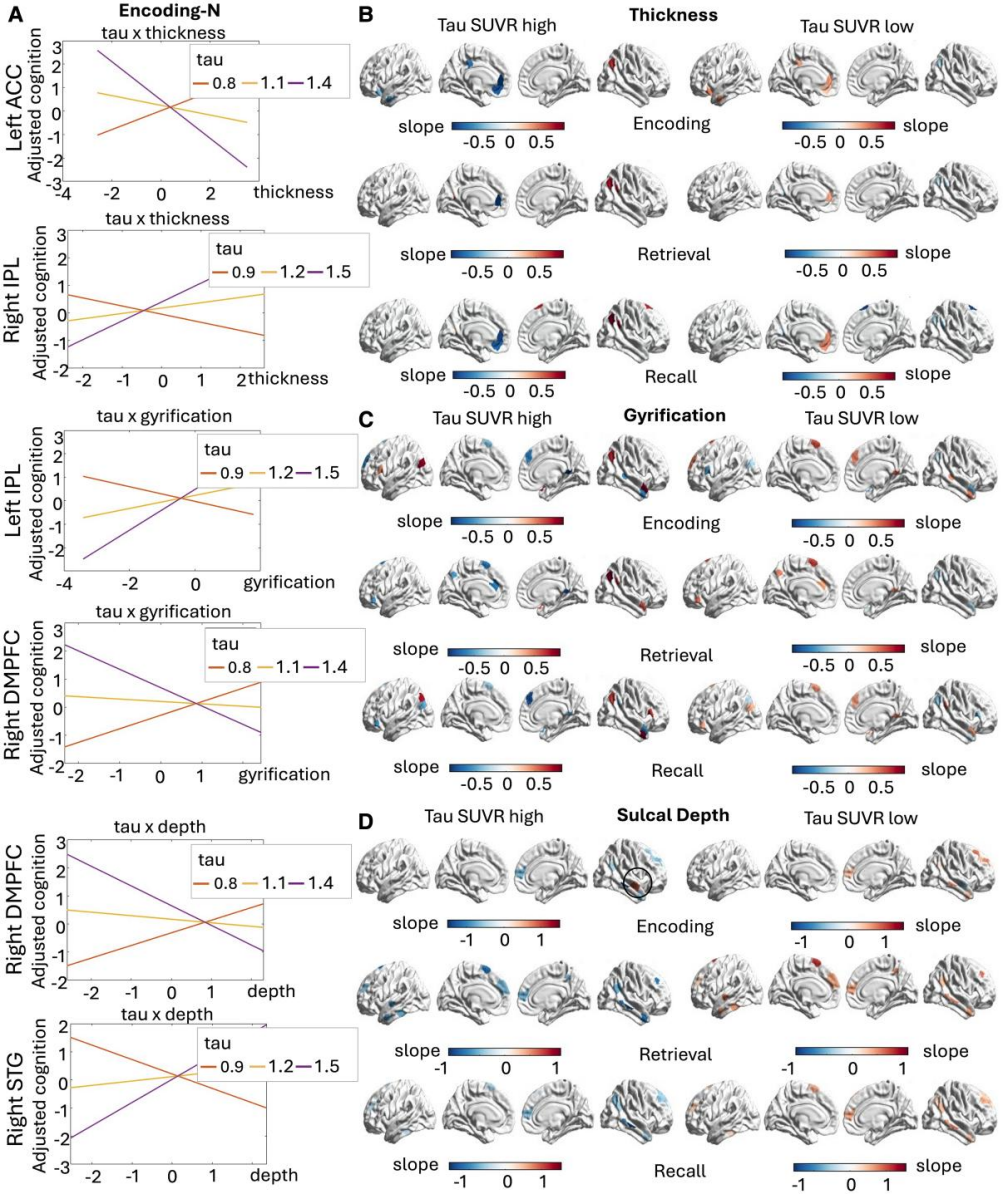
**Structural alterations in relation to memory performance in A– individuals.** Plot of the results of the multiple linear regression models (*n* = 150 individuals). Interaction plots of exemplificative regions predictive of encoding performance are shown (**A**). The slope values for all nodes with significant thickness × tau (**B**), gyrification × tau (**C**) and sulcal depth × tau (**D**) interactions in predicting encoding, retrieval and recall performance are shown on the brain surface. Regions marked with a circle indicate DMN parcels with opposite interaction effects. ACC, anterior cingulate cortex; DMPFC, dorsomedial prefrontal cortex; IPL, inferior parietal lobule; STG, superior temporal gyrus; SUVR, PET standard uptake values ratios.

In particular, we observed that in the presence of only minor tau deposits, thickness, gyrification and sulcal depth indexes of most DMN regions were positively related to encoding, retrieval and recall performance (see Fig. 3). These patterns suggest that preserved GM volume and cortical complexity are beneficially related to behaviour.

On the other hand, more variable patterns were observed in participants presenting high levels of tau deposits. For instance, only the thickness of regions located in the right hemisphere (especially the right IPL) were found positively related to encoding, retrieval and recall (Fig. 3 B) while the left MTG, left IFC, left ACC regions displayed negative associations instead (Fig. 3 B).

Similarly, the bilateral DLPFC, bilateral IPL and right STG presented positive associations between gyrification and encoding/recall scores, whereas medial structures presented a negative association instead (bilateral dorsomedial prefrontal cortex (DMPFC), left precuneus, left ACC, left IFC, see Fig. 3 C).

Finally, sulcal depth measures showed opposite relationships with encoding, retrieval and recall performance in individuals A– with lower tau burden (A–T–) compared with those participants with higher tau pathology (A–T+) (see Fig. 3 D), suggesting a possible modulatory role of age-related tauopathy in affecting cortical morphology. Of note, the only exception was observed for the right STG in the A–T+ group (marked with a circle) that presented a positive association between sulcal depth and encoding performance. As shallower sulcal depth of the temporal cortices is characteristic of a more advanced stage of pathology,^37^ this result further validates that more preserved morphology of this region favourably influences encoding capacities.

In summary, several DMN regions were found to be associated with baseline memory performance in A– individuals. While all those participants were cognitively intact (MMSE > 26), they showed variability in their degree of regional tau burden. For those with low tau levels, all morphology measures were correlated positively with all indices of memory performance. On the other hand, individuals with higher levels of tau burden showed negative associations between thickness, gyrification and sulcal depth—especially in medial structures—with encoding, retrieval and recall performance. Of note, while several DMN regions were predictive of memory performance in A– individuals, FPN structures showed almost no relationship (see Supplementary Materials, section 4).

All models’ statistics are available in Supplementary Table 2 in the Supplementary Materials.

### Baseline structural alterations as predictors of future cognitive decline

For the A+ group, no significant results on the relationship between structural alteration and memory decline survived FDR correction.

On the other hand, few regions emerged as predictors in the A– group with increasing regional tau deposits. For instance, higher baseline sulcal depth of the right ACC was associated with better encoding performance at the 2-year follow-up in individuals with higher tau deposits in this region (see Supplementary Fig. 2 B). The opposite relationship was instead observed in those with lower tau deposits.

For retrieval abilities, the gyrification of the left MTG and left PO was negatively associated with performance at the 2-year follow-up, but presented a slight positive association in the individuals with low tau deposits in these regions (see Supplementary Fig. 2 C).

Finally, higher thickness and sulcal depth in some left frontal and bilateral temporal regions (specifically, left DLPFC, left ACC, left IPL and bilateral MTG) were associated with better encoding and recall performance at the two year follow-up in individuals who presented higher tau deposits at baseline within these regions (see Supplementary Fig. 2 D). Only a portion of the right MTG presented a negative association for thickness in predicting future encoding performance (marked by a circle in Supplementary Fig. 2 D).

In summary, we confirmed the protective role of greater thickness and sulcal depth in guaranteeing more preserved encoding and recall abilities in A– individuals with high tau burden at baseline. All models’ statistics are available in Supplementary Table 3 in the Supplementary Materials.

### Prediction of future tau accumulation based on baseline structural alterations

**Figure 4:**
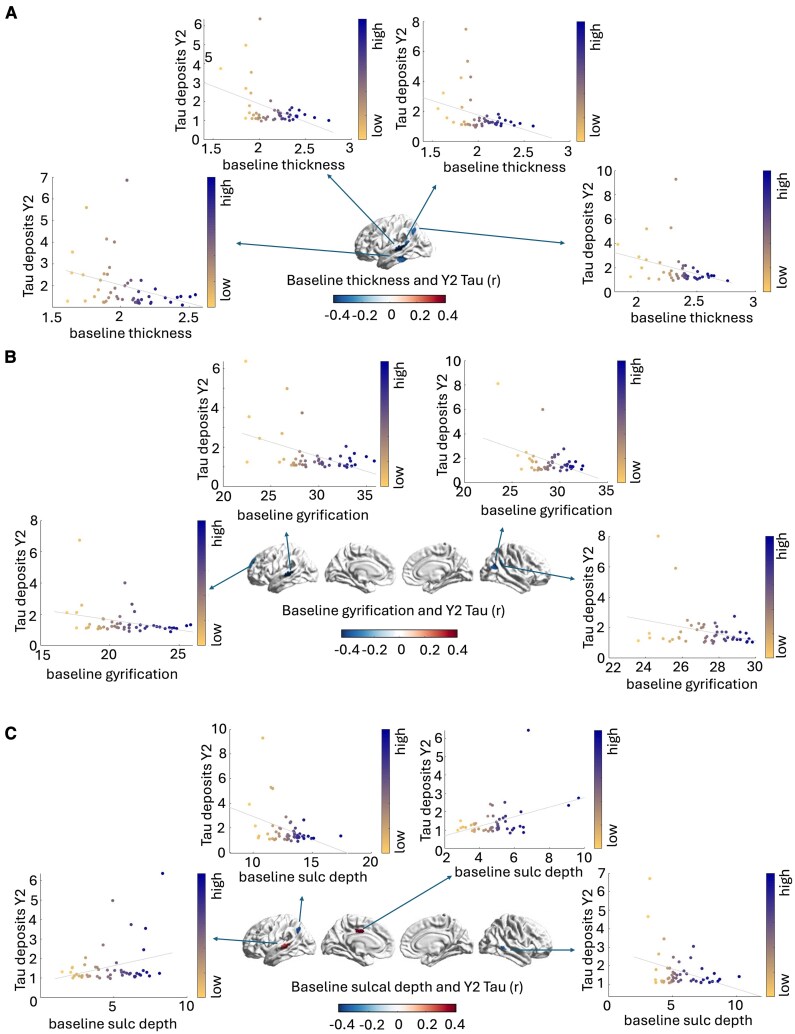
**Associations between baseline structural measures and future tau burden in A+**  **individuals.** Results of Pearson’s correlation analyses (*n* = 49 individuals). DMN regions presenting baseline thickness (**A**), gyrification (**B**) and sulcal depth measures (**C**) associated with tau SUVRs at 2-year follow-up. A+, amyloid positive; DMN, default mode network; SUVR, standard uptake values ratio; Y2, year 2 follow-up.

In the A+ group, we observed that higher baseline thickness in temporal and parietal regions of the left hemisphere was associated with lower tau deposits at the two year follow-up (left inferior temporal gyrus-ITG: *r* = −0.33, *p* = 0.021, left IPL: *r =* −0.33, *p* = 0.02, two regions of the left STG: *r =* −0.41, *p* = 0.003; *r =* −0.33, *p* = 0.02) (see Fig. 4 A). Similarly, higher gyrification values at baseline in left fronto-temporal regions and right parietal regions were associated with lower tau accumulation at the two year follow-up (left STG: *r =* −0.46, p< 0.0001, left DLPFC: *r =* −0.31, *p* = 0.03, and two regions in the right IPL: *r =* −0.42, *p* = 0.0027; *r =* −0.30, *p* = 0.036) (see Fig. 4 B). A more diversified pattern was observed for sulcal depth measures, for which the greater sulcal depth of the left STG (*r =* 0.29, *p* = 0.04) and left PCC (*r =* 0.41, *p* = 0.003) was found to be associated with higher tau burden at the 2-year follow-up, opposite to the pattern observed for the left IPL (*r =* −0.37, *p* = 0.008) and right MTG (*r =* −0.34, *p* = 0.016) (see Fig. 4 C).

**Figure 5:**
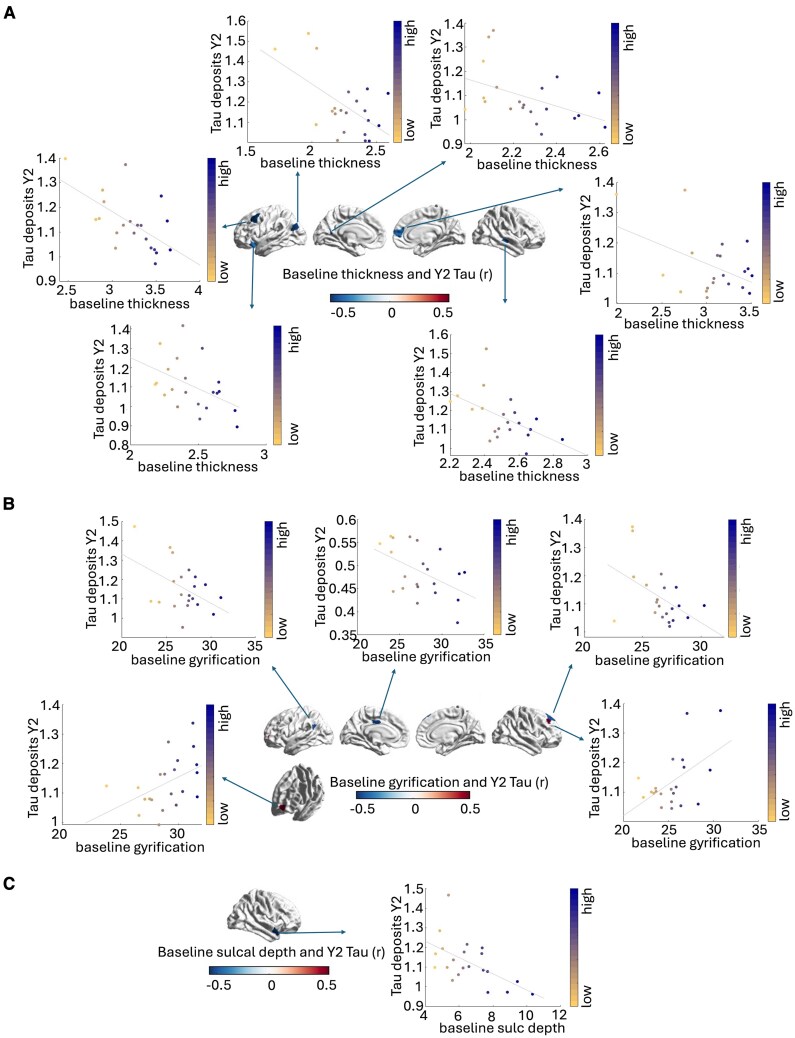
**Associations between baseline structural measures and future tau burden in A– individuals.** Results of Pearson’s correlation analyses (*n* = 21 individuals). DMN regions presenting baseline thickness (**A**), gyrification (**B**) and sulcal depth measures (**C**) associated with tau SUVRs at 2-year follow-up (Y2). A-, amyloid negative; DMN, default mode network; SUVR, standard uptake values ratio.

In summary, greater baseline thickness, gyrification and sulcal depth were associated with reduced tau build-ups at the 2-year follow-up. In the A– group, we observed that higher baseline thickness was also associated with reduced future tau accumulation across a variety of DMN regions (left IPL: *r =* −0.57, *p* = 0.006, left DLPFC: *r =* −0.6, *p* = 0.004, left IFC: *r =* −0.45, *p* = 0.038, left precuneus: *r =* −0.44, *p* = 0.044, left MPFC: *r =* −0.52, *p* = 0.014, right DLPFC: *r =* −0.46, p = 0.034 and right MTG: *r =* −0.52, *p* = 0.015) (see Fig. 5 A). For gyrification measures, we observed that higher gyrification was associated with lower future accumulation of tau for the left IPL (*r =* −0.46, *p* = 0.035), left PCC (*r =* −0.51, *p* = 0.017), and right DLPFC (*r =* −0.49, *p* = 0.024), but not for the left IFG (*r =* 0.45, *p* = 0.039) and for a portion of the right DLPFC (*r =* 0.53, *p* = 0.014) (see Fig. 5 B). Finally, only the levels of sulcal depth within the right STG (*r =* −0.57, *p* = 0.007) was observed to be associated negatively with future tau deposits (see Fig. 5 C).

In summary, greater baseline thickness, gyrification and sulcal depth were associated with reduced tau build-ups at the 2-year follow-up. Unfortunately, however, none of the observed correlations survived FDR correction, possibly due to the small sample size (*n* A+= 49; *n* A−= 21). Early discontinuation and withdrawal information of all patients is available in the ADNI website (https://adni.loni.usc.edu/).

## Discussion

The present study aimed at investigating the relationship between structural alterations of DMN regions and memory performance as a function of amyloid status (A) and tau deposits (T). While several studies have looked at structural alterations in the Alzheimer’s disease spectrum, here we focus specifically on regions that have been primarily studied in terms of their *functional* alterations. Furthermore, we combined traditional cortical thickness measures with indices of cortical complexity (gyrification and sulcal depth) for which fewer studies have been conducted. Finally, we checked whether such structural alterations were predictive of future tau accumulation within the same regions.

We initially hypothesized that a highly damaged DMN network in the most severe patients (A+ with also hight tau burden) would have a weaker relationship with memory performance, whereas its degree of disruption would be associated with memory impairment in the milder stages of the Alzheimer’s disease continuum and in healthy controls. However, our results support a significant role of the DMN across the healthy aging to Alzheimer’s disease spectrum. In particular, the significance of our study lies in demonstrating the differential role of DMN structures in relation to memory abilities as a function of the presence and the degree of the pathology burden. Our interaction analyses revealed two main categories of associations—further divided into four possible combinations of proteinopathy—for which DMN integrity has a different relationship with memory performance. The first of these combinations, A–T– participants, represents our healthy subsample, that we observe to present positive associations between DMN structural integrity and encoding, retrieval and recall abilities. These findings support the notion that the DMN is one of the neural underpinnings of episodic memory.^41-43,45^ Interestingly, A+T+ individuals also displayed positive associations between thickness and gyrification with all memory functions. This group represents the most severe patients, with the strongest interaction observed for those with the lowest MMSE scores. As amyloid positivity facilitates thinning of the cortex^63^ and because the latter is associated with greater memory impairment,^64^ we argue that the observed positive relationship reflects a protective role of residual integrity of the cortex in supporting preservation of memory capabilities. Opposite to those results, A–T+ and A+T– individuals presented negative structure-memory relationships. These findings seem to go against the expected ‘bigger is better’ relationship between structural properties and cognitive performance.^65^ While surprising, prior reports of negative associations are present in the literature for GM volume^65^ and gyrification.^31-33^ In primary age-related tauopathy (i.e. A–T+), tau build-ups appear earlier than structural damage and remain confined to medial temporal lobe structures and cingulate regions.^8^ These same regions emerged among the ones where higher cortical thickness was associated with worse memory performance in our analyses. This might reflect neuronal swelling or specific instances where tau pathology is more marked than structural damage (thinning rate and alterations in the cortical folding), thus resulting in patterns of impaired cognitive functions in the presence of less cortical atrophy. Specific to the findings for gyrification, a prior published study suggested that increase in the cortical folding in the context of pathology might happen to favour the formation of new connections in the face of progressive atrophy.^31^ While this hypothesis is unlikely to explain the pattern of all regions, it is still worth considering as a possible explanation of the negative associations between gyrification and memory performance, highlighting the fact that increased gyrification might underlie a pathological response mechanism.^31^ Finally, the majority of the studies on sulcal morphology in Alzheimer’s disease have focused on a relatively small number of primary sulci, without detailing the changes of tertiary sulci in frontal and parietal cortices, that ontogenetically develop later and are more closely linked to interindividual differences in higher-order behaviour,^66^ and that might be of particular interest in this study since DMN regions largely overlap with associative cortices. Furthermore, there is extensive evidence on the association between sulcal depth and aging (evidence of a decrease, see for example^34,67,68^), as well as sulcal depth and pathology (shallower temporal sulci with more advanced disease stages, e.g.^37,69^). However, to the best of our knowledge, only one study has been published looking at the relationship between sulcal depth and cognition in the Alzheimer’s disease spectrum.^33^ Similar to the patterns observed in this study, Coleman and colleagues^33^ also observed both positive and negative associations between the sulcal depth of different regions with memory, language and executive function skills. However, the authors did not discuss the difference observed in the directionality of their correlational values with behaviour. While positive associations are to be expected (greater cortical complexity is linked to better behaviour, with the depth of the sulci being a direct measure of efficiency of short range connections, see^70^), negative relationships remain more challenging to explain. Still, some developmental studies also report such a negative association between the depth of tertiary sulci (which are naturally shallower) and cognitive performance.^70,71^ In these studies, similar to ours, shallower measures of certain sulci were associated with better (working) memory performance.^71^ In this respect, the depth of cortical sulci seems to vary in the cortex to maintain a sort of homeostasis between deeper and shallower sulci, possibly resulting in positive and negative associations with behaviour. Given the lack of further literature on this matter, this reasoning remains only speculative and future studies will be needed to address this issue fully.

Of note, we observed that the structural indexes of several DMN regions also remained predictive of memory performance at the 2-year follow-up, but only in the A– participants. The A–T+ group represents a particularly vulnerable population with age-related tauopathy that could be indicative of prodromal stages of different neurodegenerative disorders.^72^ In this group,^73,74^ higher baseline thickness and sulcal depth of few regions in the bilateral cingulate, MTG and left DLPFC were associated with more preserved recall performance at the 2-year follow-up. The exact opposite pattern was observed in A–T– participants. This result suggests that in the presence of tauopathy, greater structural integrity is associated with future better outcomes. Conversely, in healthy individuals, efficient cognitive performance might depend on greater neural computation efficiency^65^ (the capacity to maintain efficient functioning at lower cost, i.e. reduced regional volume).

A last observation within our findings is the left hemisphere preponderance of brain regions the thickness of which was significantly associated with baseline memory performance in A+ individuals. This appears in line with prior reports of asymmetrical cortical thinning, that suggests that the left hemisphere is more affected by the pathology.^75-77^ Evidence of greater tau accumulation in the left hemisphere has also recently been reported, and this was linked to earlier pathological onset and poorer prognosis.^78^

Finally, for several DMN regions in both A+ and A– individuals, higher thickness and gyrification at baseline was associated with lower future tau accumulation in the same regions. This pattern appears in line with the brain reserve hypothesis that postulates that individuals with a higher physical threshold (e.g. greater preserved GM and cortical complexity) have greater resistance against future tau accumulation. A recent study has also suggested that individuals with low brain reserve were less resilient to pathological tau, although this was tested more indirectly and without controlling for future tau build-ups.^79^ The only exceptions to this trend appeared to be regions in the left PCC and MTG in A+ participants, and in the left IFC and right DLPFC in A– individuals. For these regions, greater cortical folding (sulcal depth and gyrification respectively) was associated with greater future tau build-ups. However, caution in the interpretation of these results is needed, as they did not survive FDR correction, possibly due to the limited sample size of individuals with MRI and PET data acquisitions at both baseline and follow-up. Of note, other known demographic variables were observed to impact memory performance meaningfully, such as the negative impact of older age as opposed to the favourable effect of higher education (see Supplementary Materials).

In conclusion, our results highlight that morphological features within the DMN show different susceptibility to the proteinopathy and are predictors of baseline and longitudinal memory performance. Indeed, when the same analyses were performed on the FPN (which shows great physical proximity to the DMN), only thickness measures in A+ participants were found to be related to encoding and recall performance (see Supplementary Materials, section 4). For all the other conditions, significant relationships were sporadic and limited to very small regions within that network.

### Limitations

Several limitations need to be addressed in order to have an interpretation of the results as unbiased as possible. First of all, this study mainly focused on structural alterations of the DMN and FPN, but regions within several other resting-state networks are known to undergo degeneration in the presence of pathology (for a review see^80^). Future studies, therefore, need to assess how the relationship between morphology and behaviour changes outside the DMN and the FPN. Secondly, future research will also have to replicate our findings across different samples, since it was not possible to run additional replications in this study given the constraints in the inclusion criteria, particularly the use of EMBIC-DCB measures that so far are only available for the ADNI3 cohort. Increasing sample size would also be a recommended direction for future studies. Indeed, although the effect sizes of our models suggest an adequate sample size for this study, reproducibility of brain–behaviour associations are particularly sensitive to the numerosity of the sample.^81^ Finally, here we did not control for genetic risk factors of pathology (e.g. ε4 allele of apolipoprotein E -APOE) nor did we consider the amyloid burden along the Alzheimer’s disease continuum, but rather amyloid burden was computed as a two level categorical variable, i.e. as a positive or negative status. On a similar note, we used MMSE scores as an index of clinical severity due to its broad use in clinical settings,^82,83^ but MMSE scores might not always be sensitive enough to capture fine changes in clinical severity especially in the earliest stages of disease.^84^ While we also considered tau burden and amyloid positivity to estimate the clinical severity of the patients (together with MMSE scores), future studies might want to rely on a more extensive neuropsychological and clinical evaluation.

## Conclusions

The present study investigated the association between structural alterations and memory performance along the Alzheimer’s disease spectrum as a function of amyloid and tau burden. To the best of our knowledge, this is the first study combining all such cognitive and neural metrics in an attempt to link what is known from a diagnostic perspective and what is observed in terms of present and future cognitive impairment. Interestingly, we observed different association patterns between thickness, gyrification and sulcal depth indexes as a function of the presence, and extent, of proteinopathy burden, overall identifying discriminating patterns between A+T+/A–T– and A–T+/A+T– individuals.

## Supplementary Material

fcaf320_Supplementary_Data

## Data Availability

All data used in the analyses are made available in the ADNI’s repository (https://ida.loni.usc.edu/login.jsp). Code used for the statistical analyses is available in GitHub (https://github.com/ariannamenardi/DMN_structural_alterations_AD.git). Brain surface images were created with codes from the ENIGMA toolbox (https://enigma-toolbox.readthedocs.io/en/latest/pages/12.visualization/).
