# Supplementary material for: Cortical morphology changes in default mode network regions as predictors of cognitive decline in relation to amyloid and tau deposits
Source: Brain Commun. 2025 Aug 28;7(5):fcaf320. doi: 10.1093/braincomms/fcaf320 (PMC12418381; doi:10.1093/braincomms/fcaf320)
Supplement: fcaf320_Supplementary_Data [file fcaf320_supplementary_data.pdf]

Supplementary Material

1. CAT12 Quality Check

Weighted Image Quality Rating (wIQR) scores from CAT12 (https://neuro-jena.github.io/cat12-help/#qc) were used to ensure that all participants had MRI images of sufficient quality. For all our sample, wIQR scores suggest images of sufficient, good or excellent data quality (wIQR 1.86-4.07, mean= 2.45±0.38). Supplementary Figure 1 reports the histogram distribution of wIQR scores of our sample of participants.

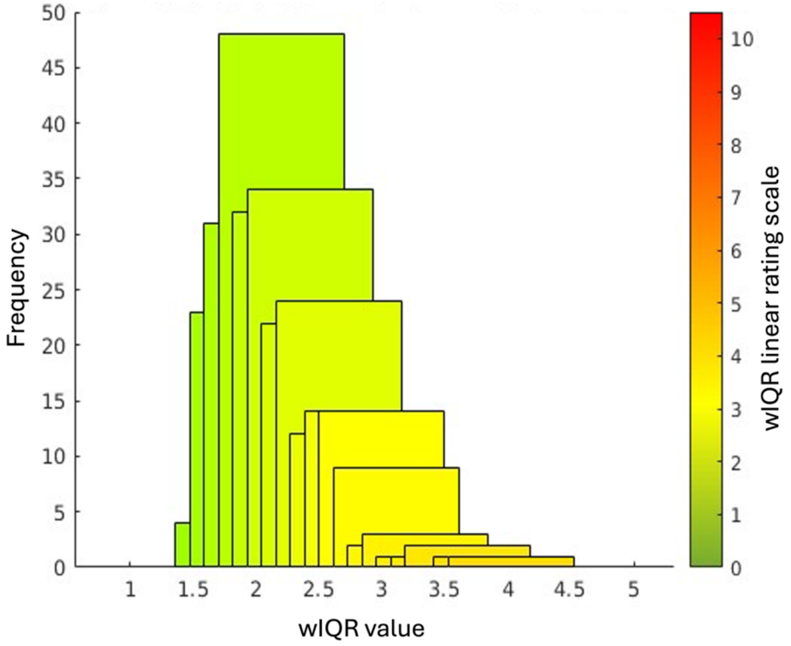

Supplementary Figure 1. wIQR scores distribution in our sample.
wIQR = Weighted Image Quality Rating

2. Association/dissociation of the investigated measures

We compared surface-based measures with the more "traditional" gray matter volume (GMV). GMV was computed by means of default settings in CAT12 software (http://www.neuro.uni-

jena.de/cat), implemented in SPM12 (http://www.fil.ion.ucl.ac.uk/spm), which was run in MATLAB 2023a (MathWorks, Natick, MA). Repeated measure ANOVA was then used to compared GMV with the other metrics (thickness, gyrification and sulcal depth). We observed significant differences among all four metrics ($F = 203522$, $p<0.0001$, all post-hoc analyses $p<0.0001$). As per the association/dissociation among the metrics (e.g., sulcal depth, gyrification, thickness) the variance inflation factor (VIF) was already computed within all models in the main text, which always resulted to be VIF~1. This suggests no risk of multicollinearity between measures at the single parcel level and further confirms the individual contribution as a predictor. Nevertheless, we also directly tested the Spearman correlation between these measures and indeed found them not to be correlated (sulcal depth by gyrification: $r = -0.03$, $p = 0.6$; thickness by gyrification: $r = -0.11$, $p = 0.06$; thickness by sulcal depth; $r = -0.06$, $p = 0.33$). On the other hand, grey matter volume was significantly associated with thickness ($r = 0.43$, $p<0.0001$) and sulcal depth ($r = 0.43$, $p<0.0001$) but not with gyrification ($r = 0.09$, $p = 0.13$). This is in line with prior studies reporting a strict link between thickness and grey matter volume, as the latter is a less specific measure that captures both thickness and cortical folding patterns [1,2]. For these reasons, both studies argue in favor of surface-based estimates, rather than volume-based estimates, as more sensitive to AD signature changes in the cortex.

Finally, we compared measures' sensitivity (in terms of their association, i.e., Spearman correlation) to tau burden levels and MMSE scores. For the former, we observed a significant negative association with thickness ($r = -0.27$, $p< 0.0001$) and gyrification ($r = -0.18$, $p = 0.002$), but not with sulcal depth ($r = -0.06$, $p = 0.3$). The same pattern was observed in relation to MMSE scores, with significant Spearman correlations with thickness ($r = 0.33$, $p<0.0001$), gyrification ($r = 0.21$, $p<0.0001$), but not sulcal depth ($r = 0.11$, $p = 0.06$). These results suggest that thickness

and gyrification might be more sensitive than sulcal depth to variations in tau burden and general cognitive performance in the AD spectrum.

3. DMN regions predictive of memory decline at 2 years follow-up

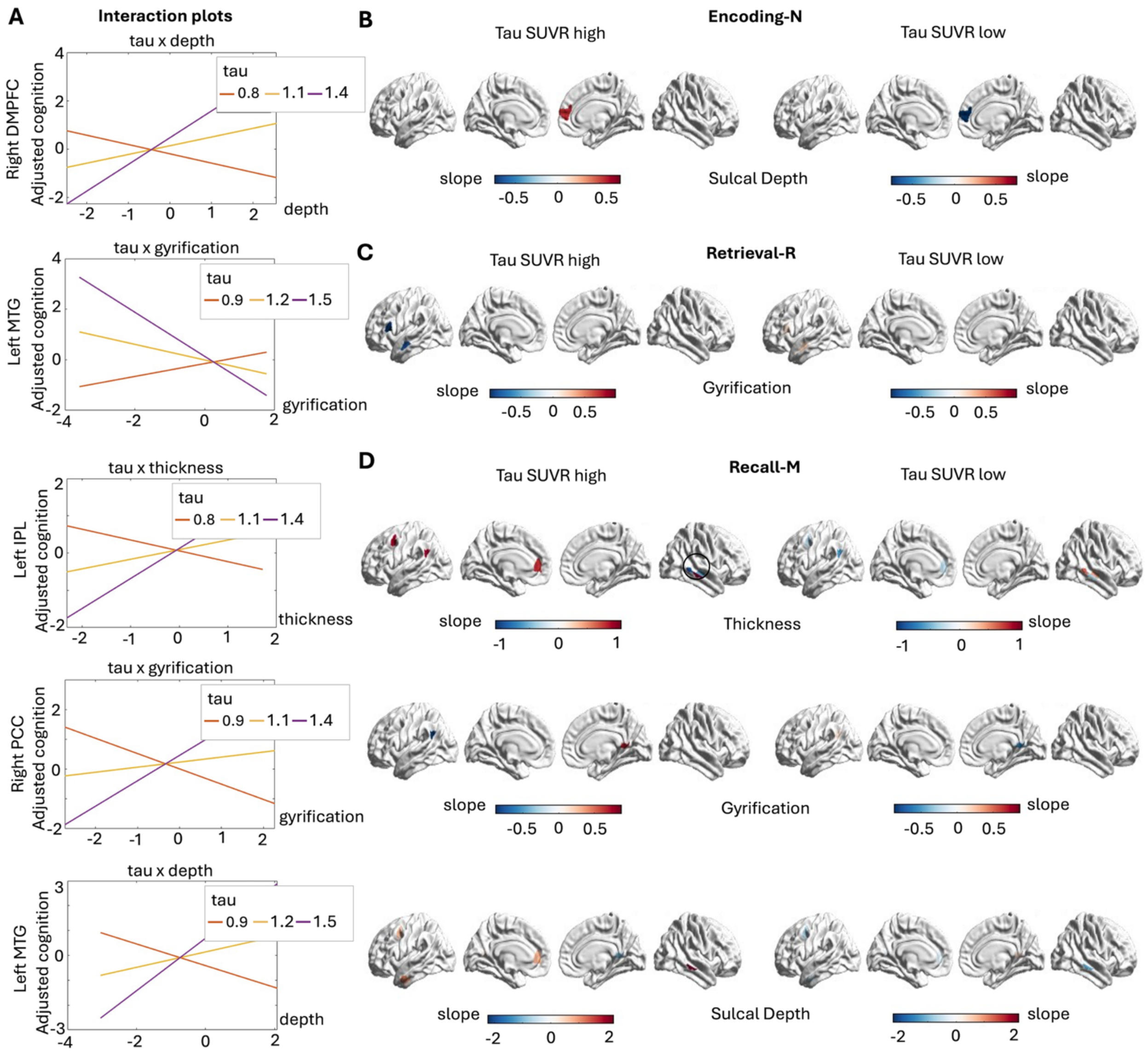

Supplementary Figure 2. Structural alterations in relation to memory decline at 2 years follow-up in A- individuals. Plot of the results of the multiple linear regression models (n = 98 individuals). Interaction plots of exemplificative regions for encoding, retrieval and recall performance are shown (**A**). The slope values for all nodes with significant tau interaction are shown on the brain surface for encoding (**B**), retrieval (**C**) and recall (**D**) performance. The color code reflects the directionality and steepness of the interaction slope (i.e., red colors indicate a positive interaction, whereas blue colors indicate a negative slope).
DMPFC = dorsomedial prefrontal cortex, MMSE = mini mental state examination, MTG = middle temporal gyrus, IPL = inferior parietal lobule, PCC = posterior cingulate cortex, SUVR = PET standard uptake values ratio.

4. Control analyses on the Frontoparietal Network

To control for the specificity of the DMN findings discussed in the main text of the manuscript, we run the same analyses on a neighbouring network, the frontoparietal (FPN) network. While we observed a similar pattern to that of the DMN, in terms of directionality of the effects, the thickness of FPN regions was the main significant predictor of baseline memory performance (encoding, retrieval and recall) in A+ individuals (see Supplementary Figure 3 and Supplementary Table 4). On the other hand, cortical complexity measures (gyrification and sulcal depth) did not emerge as particular strong predictors (see Supplementary Figure 3 and Supplementary Table 4), with limited and small portions of the lateral frontal and temporal cortices displaying a significant interaction.

Overall, cortical morphology measures of the FPN were observed to be more strongly associated with baseline memory performance of A+ individuals rather than A-. For this latter group, only few small regions in the dorsolateral prefrontal cortex emerged as significant predictors (see Supplementary Figure 4 and Supplementary Table 5).

As per the ability to predict memory decline at 2 years follow-up, we again observed very little contribution by small portions of the dorsolateral cortex and parietal lobule in predicting retrieval and recall performance in A- individuals. No significant results were found for encoding abilities nor for A+ individuals (see Supplementary Figure 5 and Supplementary Table 6).

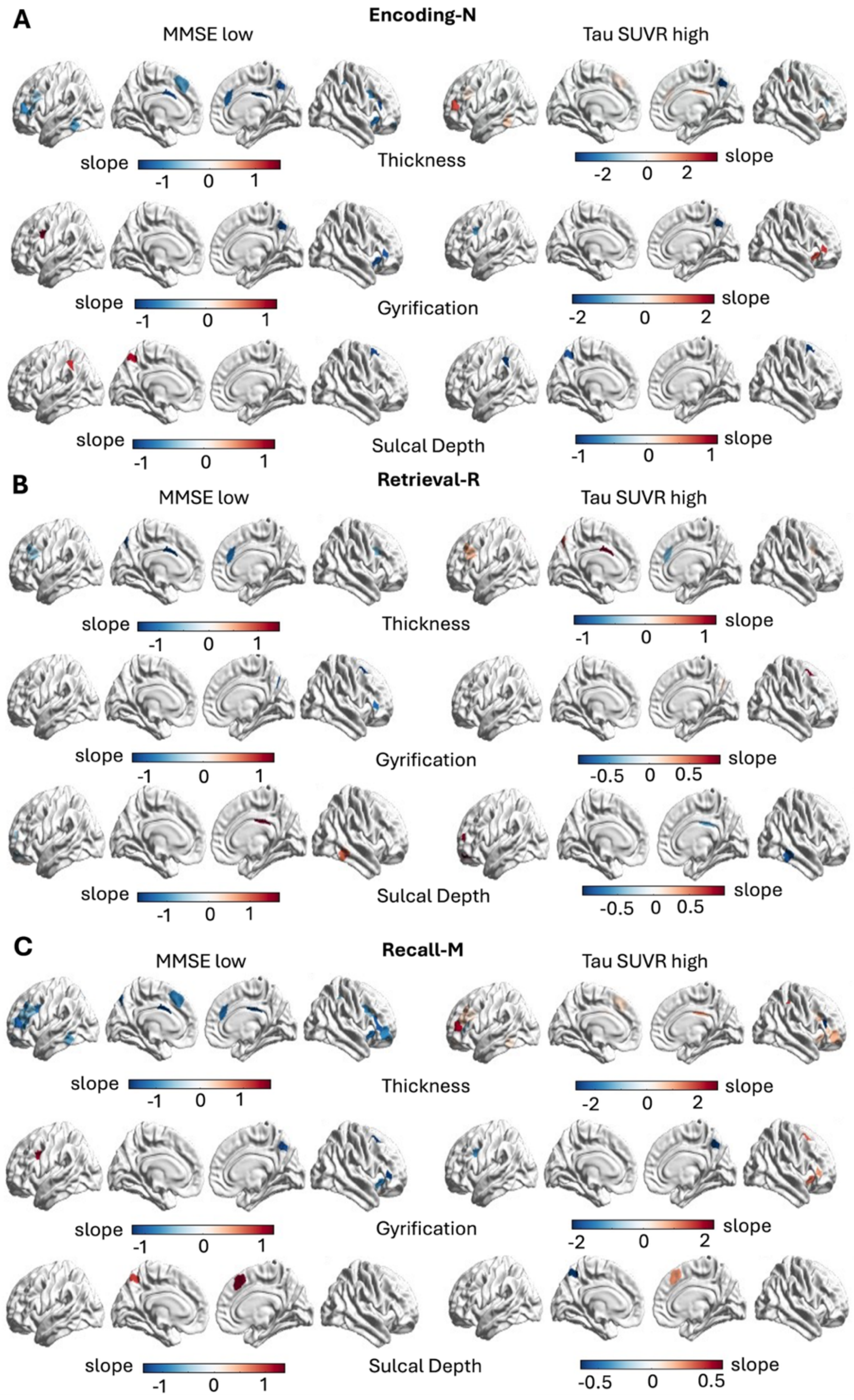

Supplementary Figure 3. FPN cortical morphology measures in relation to memory performance in A+ individuals. Plot of the results of the multiple linear regression models (n = 129 individuals). The slope values for all nodes with significant interaction with MMSE scores for encoding (**A**), retrieval (**B**) and recall (**C**) are shown on the brain surface. The color code reflects the directionality and steepness of the interaction slope (i.e., red colors indicate a positive interaction, whereas blue colors indicate a negative slope).
MMSE = Mini Mental State Examination, SUVR = standard uptake value ratio

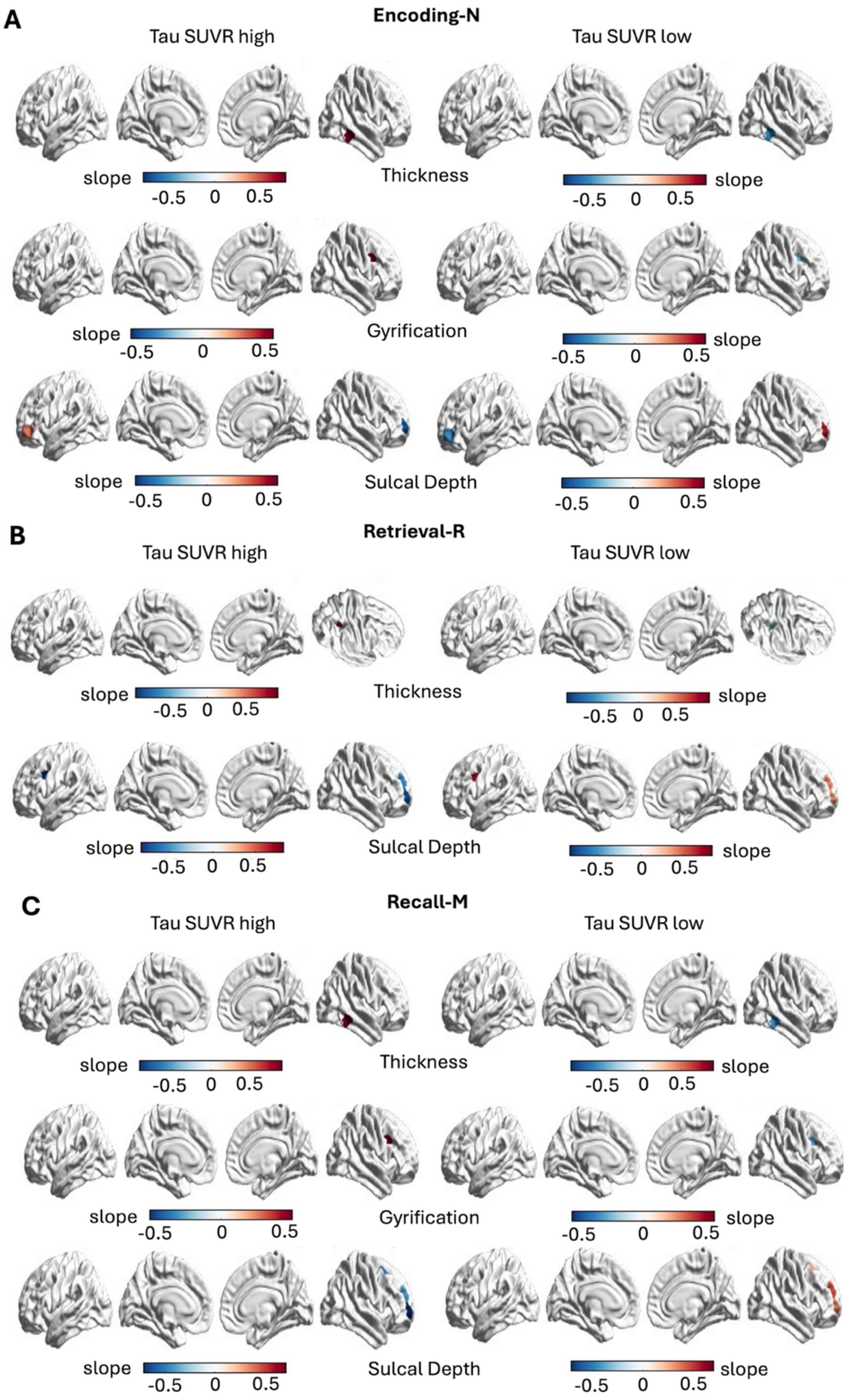

Supplementary Figure 4. FPN cortical morphology measures in relation to memory performance in A- individuals. Plot of the results of the multiple linear regression models (n = 138 individuals). The slope values for all nodes with significant interaction with tau SUVR scores for encoding (**A**), retrieval (**B**) and recall (**C**) are shown on the brain surface. The color code reflects the directionality and steepness of the interaction slope (i.e., red colors indicate a positive interaction, whereas blue colors indicate a negative slope).
SUVR = standard uptake value ratio

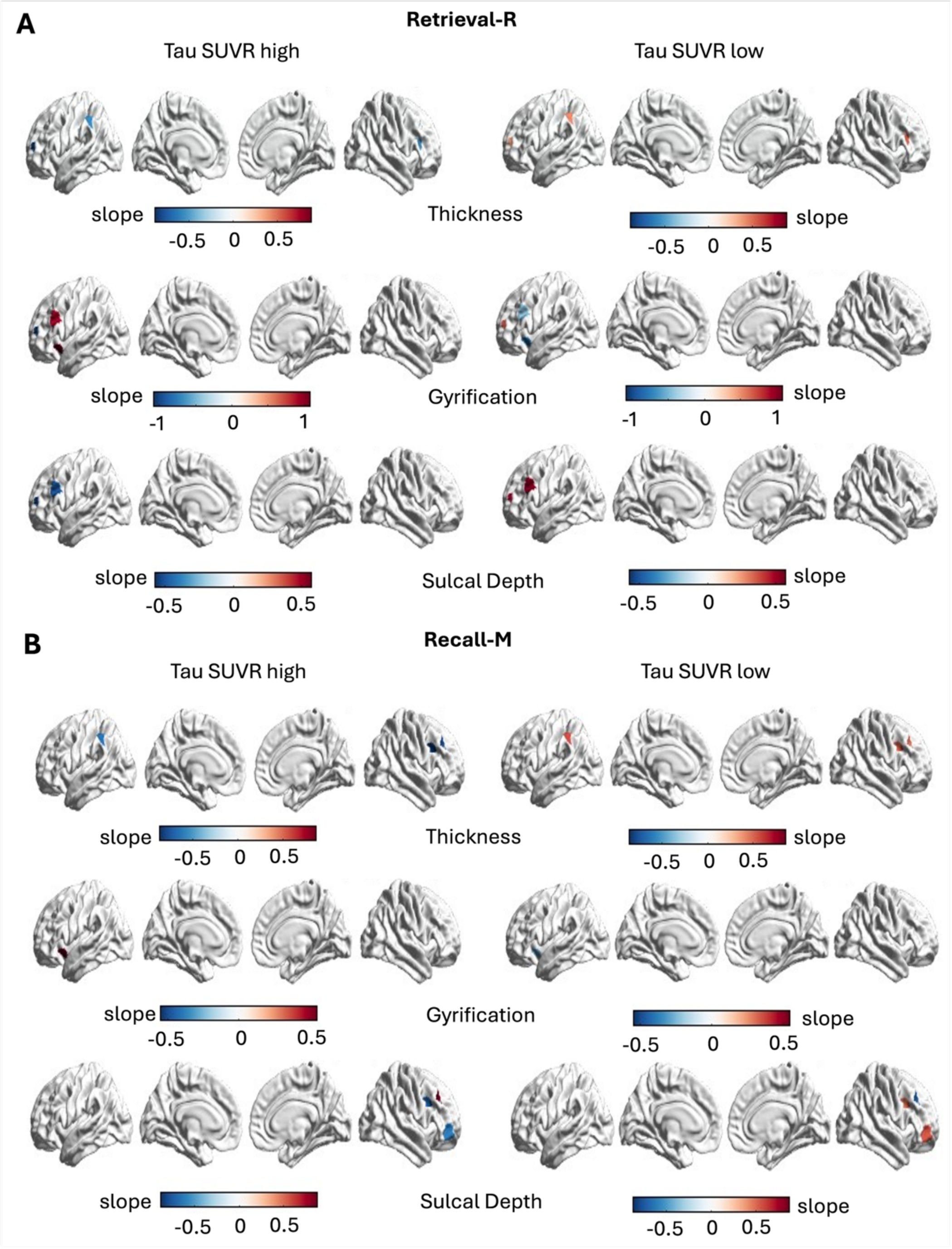

Supplementary Figure 5. FPN structural alterations in relation to memory decline at 2 years follow-up in A- individuals. Plot of the results of the multiple linear regression models (n = 89 individuals). The slope values for all nodes with significant tau interaction are shown on the brain surface for encoding (**A**), retrieval (**B**) and recall (**C**) performance. The color code reflects the directionality and steepness of the interaction slope (i.e., red colors indicate a positive interaction, whereas blue colors indicate a negative slope).
SUVR = standard uptake value ratio

5. Supplementary tables

Supplementary Table 1. Models' statistics of the significant association between DMN structural measures and MMSE scores in predicting baseline encoding, retrieval and recall performance in A+ individuals.

**Amyloid Positive - Baseline**								
**Encoding- N**			**Retrieval- R**			**Recall- M**		
**ROI**	**Model**	**Predictors**	**ROI**	**Model**	**Predictors**	**ROI**	**Model**	**Predictors**
149<br>left<br>MTG	$F_{(16,123)}$=5.03<br>p<0.0001<br>R^2=0.43	thickxMMSE<br>b=0.22,<br>p=0.016<br>AGE<br>b=-0.28,<br>p=0.001<br>TAU<br>b= -0.32,<br>p=0.002	149<br>left MTG	$F_{(16,122)}$=6.1<br>p<0.0001<br>R^2=0.48	depthxMMSE<br>b=-0.33<br>p=0.004<br>thickxMMSE<br>b=0.22<br>p=0.013<br>depthxEDU<br>b=0.19,<br>p=0.028<br>thickxEDU<br>b=0.22,<br>p=0.013<br>AGE<br>b=-0.25<br>p=0.003<br>TAU<br>b=-0.22<br>p=0.025	149<br>left<br>MTG	$F_{(16,123)}$=7.57<br>p<0.0001<br>R^2=0.53	thickxMMSE<br>b=0.3<br>p=0.0004<br>depthxEDU<br>b=0.19,<br>p=0.022<br>AGE<br>b=-0.2<br>p=0.01<br>TAU<br>b=-0.35<br>p=0.0002
155<br>left<br>MTG	$F_{(16,123)}$=4.54<br>p<0.0001<br>R^2=0.41	gyrixMMSE<br>b=0.21<br>p=0.037<br>AGE<br>b=-0.29<br>p=0.001<br>TAU<br>b=-0.26<br>p=0.009	150<br>left MTG	$F_{(16,118)}$=6.06<br>p<0.0001<br>R^2=0.49	thickxMMSE<br>b=0.22,<br>p=0.049<br>gyrixMMSE<br>b=0.23,<br>p=0.049<br>depthxEDU<br>b=-0.16,<br>p=0.035<br>AGE<br>b=-0.25,<br>p=0.0007<br>TAU<br>b=-0.26,<br>p=0.005	150<br>left MTG	$F_{(16,122)}$=8.08<br>p<0.0001<br>R^2=0.55	thickxMMSE<br>b=0.24,<br>p=0.034<br>depthxEDU<br>b=-0.17,<br>p=0.025<br>AGE<br>b=-0.19,<br>p=0.009<br>TAU<br>b=-0.39,<br>p<0.0001
156<br>left<br>MTG	$F_{(16,123)}$=4.49<br>p<0.0001<br>R^2=0.40	depthxMMSE<br>b=-0.26<br>p=0.025<br>AGE<br>b=-0.19<br>p=0.029<br>TAU<br>b=-0.29<br>p=0.005	157<br>left<br>MTG	$F_{(16,122)}$=4.64<br>p<0.0001<br>R^2=0.41	thickxMMSE<br>b=0.29<br>p=0.002<br>AGE<br>b=-0.22<br>p=0.01<br>TAU<br>b=-0.27<br>p=0.02	154<br>left<br>MTG	$F_{(16,123)}$=7.17<br>p<0.0001<br>R^2=0.52	gyrixMMSE<br>b=0.22<br>p=0.037<br>AGE<br>b=-0.18<br>p=0.023<br>TAU<br>b=-0.4<br>p<0.0001
157<br>left	$F_{(16,123)}$=4.98	gyrixMMSE<br>b=0.22	158<br>left	$F_{(16,122)}$=4.79<br>p<0.0001	gyrixMMSE<br>b=0.17	156<br>left	$F_{(16,123)}$=5.53<br>p<0.0001	depthxMMSE<br>b=-0.23

MTG	p<0.0001<br>R^2=0.43	p=0.006<br>AGE<br>b=-0.31<br>p=0.0003<br>TAU<br>b=-0.24<br>p=0.038	MTG	R^2=0.42	p=0.045<br>thickxMMSE<br>b=0.28<br>p=0.005<br>depthxEDU<br>b=-0.15,<br>p=0.041<br>TAU<br>b=-0.26<br>p=0.008	MTG	R^2=0.45	p=0.036<br>TAU<br>b=-0.34<br>p=0.0008
158<br>left<br>MTG	$F_{(16,123)}$=4.51<br>p<0.0001<br>R^2=0.40	depthxMMSE<br>b=0.31<br>p=0.025<br>thickxMMSE<br>b=0.22<br>p=0.026<br>depthxEDU<br>b=-0.32,<br>p=0.009<br>AGE<br>b=-0.23<br>p=0.01<br>TAU<br>b=-0.27<br>p=0.005	163<br>left<br>IPL	$F_{(16,122)}$=4.63<br>p<0.0001<br>R^2=0.41	depthxMMSE<br>b=-0.26<br>p=0.017	157<br>left<br>MTG	$F_{(16,123)}$=6.71<br>p<0.0001<br>R^2=0.50	thickxMMSE<br>b=0.22<br>p=0.007<br>gyrixMMSE<br>b=0.18<br>p=0.015<br>AGE<br>b=-0.21<br>p=0.009<br>TAU<br>b=-0.31<br>p=0.004
162<br>left<br>IPL	$F_{(16,123)}$=4.95<br>p<0.0001<br>R^2=0.43	gyrixMMSE<br>b=0.16<br>p=0.027<br>AGE<br>b=-0.34<br>p=0.0002<br>TAU<br>b=-0.34<br>p=0.001	164<br>left<br>IPL	$F_{(16,122)}$=3.43<br>p<0.0001<br>R^2=0.34	thickxMMSE<br>b=0.22<br>p=0.038<br>AGE<br>b=-0.20<br>p=0.036	158<br>left<br>MTG	$F_{(16,123)}$=6.55<br>p<0.0001<br>R^2=0.5	thickxMMSE<br>b=0.30<br>p=0.0006<br>depthxMMSE<br>b=0.34<br>p=0.007<br>depthxEDU<br>b=-0.32,<br>p=0.003<br>TAU<br>b=-0.32<br>p=0.0002
163<br>left<br>IPL	$F_{(16,123)}$=4.03<br>p<0.0001<br>R^2=0.38	thickxMMSE<br>b=0.23<br>p=0.041<br>AGE<br>b=-0.27<br>p=0.003	166<br>left<br>OFC	$F_{(16,122)}$=4.01<br>p<0.0001<br>R^2=0.38	gyrixMMSE<br>b=0.25<br>p=0.013	160<br>left<br>IPL	$F_{(16,122)}$=8.66<br>p<0.0001<br>R^2=0.57	thickxMMSE<br>b=0.17<br>p=0.048<br>depthxTAU<br>b=0.18<br>p=0.019<br>gyrixEDU<br>b=-0.2,<br>p=0.005<br>AGE<br>b=-0.16<br>p=0.044
165<br>left IPL	$F_{(16,123)}$=4.43<br>p<0.0001<br>R^2=0.4	thickxMMSE<br>b=0.19,<br>p=0.047<br>AGE<br>b=-0.3,<br>p=0.0007	169<br>left<br>VMPFC	$F_{(16,121)}$=4.27<br>p<0.0001<br>R^2=0.39	thickxMMSE<br>b=0.26<br>p=0.008	161<br>left<br>IPL	$F_{(16,123)}$=6.09<br>p<0.0001<br>R^2=0.48	depthxMMSE<br>b=0.21<br>p=0.028<br>thickxMMSE<br>b=0.18<br>p=0.032<br>thickxEDU<br>b=-0.26,

								p=0.007 depthxEDU b=-0.22, p=0.024 TAU b=-0.34 p=0.0002
166 left OFC	$F_{(16,123)}$=5 p<0.0001 R^2=0.43	gyrixMMSE b=0.21 p=0.024 thick b=0.19, p=0.019 AGE b=-0.24 p=0.008	170 left PO	$F_{(16,121)}$=5.57 p<0.0001 R^2=0.46	thickxMMSE b=0.22 p=0.01 depth b=0.28, p=0.001	163 left IPL	$F_{(16,123)}$=5.5 p<0.0001 R^2=0.45	thickxMMSE b=0.26 p=0.017
167 left OFC	$F_{(16,123)}$=5 p<0.0001 R^2=0.43	thickxMMSE b=0.24 p=0.004 AGE b=-0.25 p=0.004	171 left MFC	$F_{(16,122)}$=4.58 p<0.0001 R^2=0.41	thickxMMSE b=0.20 p=0.02 depthxEDU b=0.18, p=0.041 AGE b=-0.19 p=0.031	164 left IPL	$F_{(16,123)}$=5.5 p<0.0001 R^2=0.46	thickxMMSE b=0.26 p=0.007 TAU b=-0.42 p=0.002
168 left VMPFC	$F_{(16,123)}$=5.42 p<0.0001 R^2=0.45	depthxMMSE b=0.27 p=0.003 gyrixMMSE b=0.22 p=0.039 thickxMMSE b=0.22 p=0.01 AGE b=-0.18 p=0.023 TAU b=-0.34 p=0.001	175 left PO	$F_{(16,122)}$=5.4 p<0.0001 R^2=0.45	depthxMMSE b=-0.21 p=0.031 depthxEDU b=0.23, p=0.013 gyri b=0.2, p=0.018 thick b=0.26, p=0.011	165 left IPL	$F_{(16,123)}$=5.1 p<0.0001 R^2=0.43	thickxMMSE b=0.23 p=0.013 AGE b=-0.2 p=0.02
170 left PO	$F_{(16,123)}$=4.21 p<0.0001 R^2=0.39	thickxMMSE b=0.19 p=0.035 AGE b=-0.23 p=0.011	180 left DLPFC	$F_{(16,119)}$=5.21 p<0.0001 R^2=0.45	thickxMMSE b=0.23, p=0.032 AGE b=-0.17, p=0.04	166 left OFC	$F_{(16,123)}$=6.33 p<0.0001 R^2=0.49	gyrixMMSE b=0.27 p=0.002
174 left ACC	$F_{(16,123)}$=6.57 p<0.0001 R^2=0.5	thickxMMSE b=0.33 p=0.001 gyrixMMSE b=0.18 p=0.029 depthxEDU b=-0.23, p=0.002 thickxTAU	181 left DLPFC	$F_{(16,121)}$=4.38 p<0.0001 R^2=0.40	gyrixMMSE b=0.22 p=0.019	167 left OFC	$F_{(16,123)}$=6.09 p<0.0001 R^2=0.48	thickxMMSE b=0.25 p=0.002 TAU b=-0.3 p=0.026

		b=0.26<br>p=0.002<br>depthxTAU<br>b=0.43,<br>p=0.023<br>AGE<br>b=-0.16<br>p=0.028						
175<br>left<br>PO	$F_{(16,123)}$=6.14<br>p<0.0001<br>R^2=0.48	depthxMMSE<br>b=-0.24<br>p=0.011<br>AGE<br>b=-0.27<br>p=0.001<br>TAU<br>b=-0.31<br>p=0.01	184<br>left<br>DLPFC	$F_{(16,121)}$=4.4<br>p<0.0001<br>R^2=0.40	gyrixMMSE<br>b=0.38<br>p=0.0005<br>AGE<br>b=-0.22<br>p=0.018	168<br>left<br>VMPFC	$F_{(16,123)}$=7.56<br>p<0.0001<br>R^2=0.54	depthxMMSE<br>b=0.22<br>p=0.007<br>thickxMMSE<br>b=0.26<br>p=0.0009<br>TAU<br>b=-0.37<br>p=0.0001
176<br>left<br>MFC	$F_{(16,123)}$=5.94<br>p<0.0001<br>R^2=0.48	thickxMMSE<br>b=0.25<br>p=0.02<br>gyrixTAU<br>b=-0.25,<br>p=0.016<br>AGE<br>b=-0.29<br>p=0.0007	192<br>left<br>PCC/<br>precuneus	$F_{(16,122)}$=4.1<br>p<0.0001<br>R^2=0.38	thickxMMSE<br>b=0.22<br>p=0.033<br>AGE<br>b=-0.18<br>p=0.043	169<br>left<br>VMPFC	$F_{(16,123)}$=5.65<br>p<0.0001<br>R^2=0.46	thickxMMSE<br>b=0.25<br>p=0.007<br>thickxEDU<br>b=-0.19,<br>p=0.039
178<br>left<br>DMPFC	$F_{(16,123)}$=5.23<br>p<0.0001<br>R^2=0.44	thickxMMSE<br>b=0.29<br>p=0.007<br>thickxTAU<br>b=0.40<br>p=0.003<br>AGE<br>b=-0.23<br>p=0.005	196<br>left<br>precuneus	$F_{(16,120)}$=5.51<br>p<0.0001<br>R^2=0.46	thickxMMSE<br>b=0.17<br>p=0.021<br>depthxEDU<br>b=-0.2,<br>p=0.031<br>AGE<br>b=-0.19<br>p=0.026<br>TAU<br>b=-0.33<br>p=0.005	170<br>left<br>PO	$F_{(16,123)}$=5.71<br>p<0.0001<br>R^2=0.46	thickxMMSE<br>b=0.28<br>p=0.001<br>TAU<br>b=-0.37<br>p=0.025
182<br>left<br>DLPFC	$F_{(16,123)}$=5.11<br>p<0.0001<br>R^2=0.43	thickxMMSE<br>b=0.27<br>p=0.001<br>gyrixEDU<br>b=0.18,<br>p=0.035<br>AGE<br>b=-0.30<br>p=0.001<br>TAU<br>b=-0.29<br>p=0.018	197<br>left<br>PCC	$F_{(16,122)}$=6.35<br>p<0.0001<br>R^2=0.50	depthxMMSE<br>b=-0.24<br>p=0.025<br>AGE<br>b=-0.17<br>p=0.029	171<br>left<br>MFC	$F_{(16,123)}$=6.27<br>p<0.0001<br>R^2=0.48	thickxMMSE<br>b=0.22<br>p=0.006<br>depthxEDU<br>b=0.2,<br>p=0.017<br>AGE<br>b=-0.16<br>p=0.046
184<br>left<br>DLPFC	$F_{(16,123)}$=5.03<br>p<0.0001<br>R^2=0.43	thickxMMSE<br>b=0.26<br>p=0.002<br>gyrixMMSE<br>b=0.36<br>p=0.0009	200<br>left<br>precuneus	$F_{(16,122)}$=4.57<br>p<0.0001<br>R^2=0.41	depthxMMSE<br>b=0.31<br>p=0.019<br>thickxMMSE<br>b=0.25<br>p=0.011	174<br>left<br>ACC	$F_{(16,123)}$=7.78<br>p<0.0001<br>R^2=0.54	thickxMMSE<br>b=0.32<br>p=0.0008<br>thickxTAU<br>b=0.17<br>p=0.033

		AGE<br>b=-0.38<br>p<0.0001<br>TAU<br>b=-0.36<br>p=0.004			AGE<br>b=-0.18<br>p=0.042<br>TAU<br>b=-0.37<br>p=0.003			depth<br>b=0.29,<br>p=0.0001
186<br>left<br>DLPFC	$F_{(16,123)}$=4.14<br>p<0.0001<br>R^2=0.38	depthxMMSE<br>b=0.20<br>p=0.039<br>AGE<br>b=-0.31<br>p=0.003<br>TAU<br>b=-0.30<br>p=0.017	363<br>right<br>IPL	$F_{(16,121)}$=4.41<br>p<0.0001<br>R^2=0.40	thickxMMSE<br>b=0.24<br>p=0.016	175<br>left<br>PO	$F_{(16,123)}$=8.98<br>p<0.0001<br>R^2=0.58	depthxMMSE<br>b=-0.24<br>p=0.005<br>TAU<br>b=-0.32<br>p=0.003
192<br>left<br>PCC/<br>precuneus	$F_{(16,123)}$=5.42<br>p<0.0001<br>R^2=0.45	thickxMMSE<br>b=0.29<br>p=0.003<br>gyrixEDU<br>b=0.23,<br>p=0.006<br>AGE<br>b=-0.27<br>p=0.001	366<br>right<br>IPL	$F_{(16,122)}$=5.94<br>p<0.0001<br>R^2=0.48	gyrixMMSE<br>b=0.29<br>p=0.009<br>gyrixEDU<br>b=-0.22,<br>p=0.032<br>depth<br>b=-0.18,<br>p=0.012<br>AGE<br>b=-0.16<br>p=0.029	176<br>left<br>MFC	$F_{(16,123)}$=5.68<br>p<0.0001<br>R^2=0.46	thickxMMSE<br>b=0.32<br>p=0.003<br>TAU<br>b=-0.31<br>p=0.002
197<br>left<br>PCC/<br>precuneus	$F_{(16,123)}$=4.89<br>p<0.0001<br>R^2=0.42	depthxMMSE<br>b=-0.3<br>p=0.009<br>AGE<br>b=-0.33<br>p=0.0002	373<br>right<br>MTG	$F_{(16,122)}$=4.39<br>p<0.0001<br>R^2=0.40	gyrixMMSE<br>b=-0.19<br>p=0.022<br>AGE<br>b=-0.18<br>p=0.032<br>TAU<br>b=-0.23<br>p=0.022	178<br>left<br>DMPFC	$F_{(16,123)}$=6.1<br>p<0.0001<br>R^2=0.48	thickxMMSE<br>b=0.28<br>p=0.009<br>thickxTAU<br>b=0.46<br>p=0.0005<br>depthxTAU<br>b=-0.3<br>p=0.009
200<br>left<br>precuneus	$F_{(16,123)}$=4.33<br>p<0.0001<br>R^2=0.39	thickxMMSE<br>b=0.29<br>p=0.003<br>AGE<br>b=-0.25<br>p=0.006<br>TAU<br>b=-0.29<br>p=0.025	386<br>right<br>DLPFC	$F_{(16,123)}$=4.79<br>p<0.0001<br>R^2=0.42	gyrixMMSE<br>b=0.28<br>p=0.005<br>depthxMMSE<br>b=-0.34<br>p=0.009<br>depthxEDU<br>b=0.21,<br>p=0.035<br>thick<br>b=0.2<br>p=0.04	182<br>left<br>DLPFC	$F_{(16,123)}$=5.85<br>p<0.0001<br>R^2=0.47	thickxMMSE<br>b=0.3<br>p=0.0003<br>AGE<br>b=-0.20<br>p=0.028<br>TAU<br>b=-0.26<br>p=0.026
363<br>right<br>IPL	$F_{(16,123)}$=4.16<br>p<0.0001<br>R^2=0.39	thickxMMSE<br>b=0.23<br>p=0.027<br>AGE<br>b=-0.22<br>p=0.011	397	$F_{(16,119)}$=5.35<br>p<0.0001<br>R^2=0.45	gyrixMMSE<br>b=-0.19,<br>p=0.042<br>depthxTAU<br>b=0.24,<br>p=0.03	184<br>left<br>DLPFC	$F_{(16,123)}$=6.14<br>p<0.0001<br>R^2=0.48	thickxMMSE<br>b=0.3<br>p=0.0002<br>gyrixMMSE<br>b=0.43<br>p<0.0001<br>AGE<br>b=-0.25

								p=0.005<br>TAU<br>b=-0.35<br>p=0.004
366<br>right<br>IPL	$F_{(16,123)}$=4.7<br>p<0.0001<br>R^2=0.41	gyrixMMSE<br>b=0.27<br>p=0.029<br>depthxMMSE<br>b=0.27<br>p=0.002<br>AGE<br>b=-0.23<br>p=0.006<br>TAU<br>b=-0.26<br>p=0.017	399<br>right<br>precuneus	$F_{(16,122)}$=4.56<br>p<0.0001<br>R^2=0.41	depthxMMSE<br>b=-0.25<br>p=0.045	192<br>left<br>precuneus	$F_{(16,123)}$=7.02<br>p<0.0001<br>R^2=0.52	thickxMMSE<br>b=0.35<br>p=0.0001<br>gyrixEDU<br>b=0.2,<br>p=0.009
367<br>right MTG	$F_{(16,123)}$=4.6<br>7<br>p<0.0001<br>R^2=0.41	thickxMMSE<br>b=0.26<br>p=0.012<br>gyri<br>b=0.23,<br>p=0.005<br>AGE<br>b=-0.2<br>p=0.024<br>TAU<br>b=-0.2,<br>p=0.048				196<br>left<br>PCC/<br>precuneus	$F_{(16,123)}$=5.36<br>p<0.0001<br>R^2=0.45	thickxMMSE<br>b=0.17<br>p=0.027<br>AGE<br>b=-0.18,<br>p=0.041<br>TAU<br>b=-0.49<br>p<0.0001
372<br>right<br>MTG	$F_{(16,123)}$=5.5<br>2<br>p<0.0001<br>R^2=0.45	thickxMMSE<br>b=0.25<br>p=0.028<br>depthxEDU<br>b=-0.18,<br>p=0.032<br>AGE<br>b=-0.23<br>p=0.005				197<br>left<br>PCC/<br>precuneus	$F_{(16,123)}$=7.6<br>p<0.0001<br>R^2=0.53	depthxMMSE<br>b=-0.29<br>p=0.006<br>thickxMMSE<br>b=0.19,<br>p=0.03<br>AGE<br>b=-0.19<br>p=0.016<br>TAU<br>b=-0.3<br>p=0.025
375<br>right<br>OFC	$F_{(16,123)}$=4.7<br>1<br>p<0.0001<br>R^2=0.41	thickxMMSE<br>b=0.2<br>p=0.022<br>AGE<br>b=-0.18<br>p=0.026<br>TAU<br>b=-0.31<br>p=0.025				199<br>left<br>precuneus	$F_{(16,123)}$=5.95<br>p<0.0001<br>R^2=0.47	depthxMMSE<br>b=0.22<br>p=0.023<br>thickxMMSE<br>b=0.22<br>p=0.032<br>TAU<br>b=-0.30<br>p=0.037
378<br>right<br>DLPFC	$F_{(16,123)}$=4.6<br>8<br>p<0.0001<br>R^2=0.41	gyrixMMSE<br>b=-0.27<br>p=0.006<br>depthxMMSE<br>b=-0.22<br>p=0.03<br>AGE				200<br>left<br>precuneus	$F_{(16,123)}$=5.39<br>p<0.0001<br>R^2=0.45	thickxMMSE<br>b=0.33<br>p=0.0005<br>TAU<br>b=-0.39<br>p=0.002

		b=-0.29<br>p=0.001<br>TAU<br>b=-0.34<br>p=0.001						
381<br>right<br>ACC	$F_{(16,123)}$=4.7<br>p<0.0001<br>R^2=0.41	gyrixMMSE<br>b=-0.18<br>p=0.017<br>thickxTAU<br>b=-0.24<br>p=0.032<br>gyri<br>b=0.19,<br>p=0.023<br>AGE<br>b=-0.18<br>p=0.03				363<br>right<br>IPL	$F_{(16,123)}$=5.54<br>p<0.0001<br>R^2=0.46	thickxMMSE<br>b=0.28<br>p=0.004
388<br>right<br>DLPFC	$F_{(16,123)}$=4.8<br>p<0.0001<br>R^2=0.42	thickxMMSE<br>b=0.20<br>p=0.031<br>gyrixMMSE<br>b=0.19<br>p=0.026<br>AGE<br>b=-0.31<br>p=0.0004<br>TAU<br>b=-0.32<br>p=0.003				366<br>right<br>IPL	$F_{(16,123)}$=6.22<br>p<0.0001<br>R^2=0.48	gyrixMMSE<br>b=0.36<br>p=0.002<br>depthxMMSE<br>b=0.28<br>p=0.0008<br>gyrixEDU<br>b=-0.23,<br>p=0.027<br>TAU<br>b=-0.28<br>p=0.007
389<br>right<br>DLPFC	$F_{(16,123)}$=4.0<br>3<br>p<0.0001<br>R^2=0.38	thickxMMSE<br>b=0.18<br>p=0.031<br>AGE<br>b=-0.25<br>p=0.005				367<br>right MTG	$F_{(16,123)}$=5.14<br>p<0.0001<br>R^2=0.44	thickxMMSE<br>b=0.21,<br>p=0.036<br>gyri<br>b=0.23,<br>p=0.004
390<br>right<br>DLPFC	$F_{(16,123)}$=3.8<br>9<br>p<0.0001<br>R^2=0.37	gyrixMMSE<br>b=0.25,<br>p=0.032<br>gyrixTAU<br>b=0.3,<br>p=0.022<br>AGE<br>b=-0.31,<br>p=0.0007				370<br>right<br>STG	$F_{(16,123)}$=4.37<br>p<0.0001<br>R^2=0.40	thickxMMSE<br>b=0.2<br>p=0.033<br>TAU<br>b=-0.26<br>p=0.034
395<br>right<br>PCC/<br>precuneus	$F_{(16,123)}$=3.9<br>9<br>p<0.0001<br>R^2=0.37	depthxMMSE<br>b=-0.29<br>p=0.01<br>gyrixTAU<br>b=-0.23<br>p=0.036<br>AGE<br>b=-0.23<br>p=0.008				372<br>right<br>MTG	$F_{(16,123)}$=6.14<br>p<0.0001<br>R^2=0.48	thickxMMSE<br>b=0.26<br>p=0.017<br>TAU<br>b=-0.31<br>p=0.003
396<br>right	$F_{(16,123)}$=3.9<br>6	depthxMMSE<br>b=0.29				375<br>right	$F_{(16,123)}$=6.03<br>p<0.0001	thickxMMSE<br>b=0.23

precuneus	p<0.0001<br>R^2=0.37	p=0.012<br>AGE<br>b=-0.24<br>p=0.007				OFC	R^2=0.47	p=0.008<br>depthxTAU<br>b=-0.43<br>p=0.003
397<br>right<br>precuneus	$F_{(16,123)}$=4.8<br>2<br>p<0.0001<br>R^2=0.42	depthxMMSE<br>b=-0.24<br>p=0.009<br>AGE<br>b=-0.24<br>p=0.005<br>TAU<br>b=-0.26<br>p=0.018				378<br>right<br>DLPFC	$F_{(16,123)}$=6.53<br>p<0.0001<br>R^2=0.50	gyrixMMSE<br>b=-0.27<br>p=0.003<br>depthxMMSE<br>b=-0.22<br>p=0.018<br>AGE<br>b=-0.17<br>p=0.027<br>TAU<br>b=-0.35<br>p=0.0004
398<br>right<br>PCC	$F_{(16,123)}$=4.4<br>2<br>p<0.0001<br>R^2=0.4	depthxMMSE<br>b=0.22<br>p=0.042<br>thickxTAU<br>b=0.19<br>p=0.047<br>AGE<br>b=-0.26<br>p=0.003				386<br>right<br>DLPFC	$F_{(16,123)}$=5.43<br>p<0.0001<br>R^2=0.45	thickxMMSE<br>b=0.24<br>p=0.026<br>depthxMMSE<br>b=-0.28<br>p=0.021<br>TAU<br>b=-0.37<br>p=0.006
						389<br>right<br>DLPFC	$F_{(16,123)}$=4.57<br>p<0.0001<br>R^2=0.41	thickxMMSE<br>b=0.18<br>p=0.024
						390<br>right<br>DLPFC	$F_{(16,123)}$=6.22<br>p<0.0001<br>R^2=0.49	gyrixMMSE<br>b=0.21,<br>p=0.041<br>gyrixTAU<br>b=0.25,<br>p=0.032<br>depthxTAU<br>b=-0.34,<br>p=0.013
						394<br>right<br>precuneus	$F_{(16,122)}$=5.2<br>p<0.0001<br>R^2=0.44	thickxMMSE<br>b=0.23<br>p=0.015<br>thickxEDU<br>b=-0.18,<br>p=0.026<br>gyrixEDU<br>b=0.16,<br>p=0.03
						395<br>right<br>PCC/<br>precuneus	$F_{(16,123)}$=5.29<br>p<0.0001<br>R^2=0.45	depthxMMSE<br>b=-0.31<br>p=0.003<br>gyrixTAU<br>b=-0.24<br>p=0.016<br>depthxTAU<br>b=-0.22<br>p=0.026

						397<br>right<br>precuneus	$F_{(16,123)}$=6.32<br>$p<0.0001$<br>R^2=0.49	depthxMMSE<br>b=-0.27<br>p=0.002<br>thick<br>b=0.2,<br>p=0.014<br>TAU<br>b=-0.25<br>p=0.015
						398<br>right<br>PCC	$F_{(16,122)}$=4.94<br>$p<0.0001$<br>R^2=0.43	thickxMMSE<br>b=0.17<br>p=0.045<br>thickxTAU<br>b=0.19,<br>p=0.04

ACC= anterior cingulate cortex, DMPFC= dorsomedial prefrontal cortex, DLPFC= dorsolateral prefrontal cortex, IFC= inferior frontal cortex, IPL= inferior parietal lobule, MFC= middle frontal cortex, MTG= middle temporal gyrus, OFC= orbitofrontal cortex, PCC= posterior cingulate cortex, PO= pars orbitalis, STG= superior temporal gyrus, VMPFC= ventromedial prefrontal cortex.

Supplementary Table 2. Models' statistics of the significant association between DMN structural measures and Tau SUVRs in predicting baseline encoding, retrieval and recall performance in A- individuals.

**Amyloid Negative - Baseline**								
**Encoding-N**			**Retrieval- R**			**Recall- M**		
**ROI**	**Model**	**Predictors**	**ROI**	**Model**	**Predictors**	**ROI**	**Model**	**Predictors**
149<br>left<br>MTG	$F_{(12,139)}$=2.55<br>p=0.005<br>R^2=0.20	thickxTAU<br>b=-0.45<br>p=0.035<br>AGE<br>b=-0.32<br>$p<0.0001$<br>thickxEDU<br>b=0.16,<br>p=0.024	149<br>left<br>MTG	$F_{(12,141)}$=2.4<br>p=0.007<br>R^2=0.18	depthxTAU<br>b=-0.48<br>p=0.023<br>EDU<br>b=0.20<br>p=0.004<br>AGE<br>b=-0.18<br>p=0.017	151<br>left<br>ITG	$F_{(12,139)}$=1.95<br>p=0.03<br>R^2=0.16	depthxTAU<br>b=-0.35<br>p=0.04<br>EDU<br>b=0.21<br>p=0.002
161<br>left<br>IPL	$F_{(12,138)}$=2.46<br>p=0.006<br>R^2=0.19	gyrixTAU<br>b=0.33<br>p=0.019<br>AGE<br>b=-0.27<br>p=0.0002	151<br>left<br>ITG	$F_{(12,142)}$=2.25<br>p=0.012<br>R^2=0.17	depthxTAU<br>b=-0.55<br>p=0.003<br>EDU<br>b=0.18<br>p=0.013<br>AGE<br>b=-0.19<br>p=0.02	161<br>left<br>IPL	$F_{(12,140)}$=2.12<br>p=0.02<br>R^2=0.17	gyrixTAU<br>b=0.29<br>p=0.035<br>EDU<br>b=0.19<br>p=0.006<br>AGE<br>b=-0.17<br>p=0.016
166<br>left<br>OFC	$F_{(12,138)}$=2.67<br>p=0.003<br>R^2=0.20	thickxTAU<br>b=-0.17<br>p=0.035<br>thickxEDU<br>b=0.18,<br>p=0.009<br>AGE<br>b=-0.33	156<br>left<br>MTG	$F_{(12,141)}$=3.38<br>p=0.0002<br>R^2=0.24	depthxTAU<br>b=-0.43<br>p=-0.002<br>gyrixEDU<br>b=0.18,<br>p=0.017<br>AGE<br>b=-0.23	164<br>left<br>IPL	$F_{(12,137)}$=4.19<br>$p<0.0001$<br>R^2=0.29	gyrixTAU<br>b=-0.27<br>p=0.017<br>depthxEDU<br>b=0.15<br>p=0.022<br>AGE<br>b=-0.21

		p<0.0001			p=0.004			p=0.001
169<br>left<br>VMPFC	$F_{(12,136)}$=4.41<br>p<0.0001<br>R^2=0.30	thickxTAU<br>b=-0.17,<br>p=0.048<br>depthxEDU<br>b=0.23,<br>p=0.003<br>AGE<br>b=-0.31,<br>p<0.0001	170<br>left<br>PO	$F_{(12,141)}$=3.32<br>p=0.0003<br>R^2=0.24	gyrixTAU<br>b=-0.21<br>p=0.008<br>gyrixEDU<br>b=0.16<br>p=0.043<br>AGE<br>b=-0.23<br>p=0.004	169<br>left<br>VMPFC	$F_{(12,139)}$=2.63<br>p=0.004<br>R^2=0.2	thickxTAU<br>b=-0.18,<br>p=0.035<br>EDU<br>b=0.26,<br>p<0.0001<br>AGE<br>b=-0.15,<br>p=0.029
174<br>left ACC	$F_{(12,139)}$=2.92<br>p=0.001<br>R^2=0.22	thickxTAU<br>b=-0.21,<br>p=0.005<br>AGE<br>b=-0.3,<br>p=0.0003	174<br>left<br>ACC	$F_{(12,141)}$=3.32<br>p=0.0003<br>R^2=0.24	thickxTAU<br>b=-0.22<br>p=0.005<br>EDU<br>b=0.15<br>p=0.038<br>AGE<br>b=-0.22<br>p=0.006	170<br>left<br>PO	$F_{(12,138)}$=3.21<br>p=0.0005<br>R^2=0.24	gyrixTAU<br>b=-015<br>p=0.044<br>EDU<br>b=0.25<br>p=0.0001<br>AGE<br>b=-0.14,<br>p=0.048
175<br>left<br>PO	$F_{(12,140)}$=2.52<br>p=0.005<br>R^2=0.19	gyrixTAU<br>b=0.21<br>p=0.024<br>AGE<br>b=-0.31<br>p=0.0001	177<br>left<br>ACC	$F_{(12,142)}$=3.09<br>p=0.0007<br>R^2=0.22	gyrixTAU<br>b=-0.19<br>p=0.017<br>gyrixEDU<br>b=0.17<br>p=0.012<br>AGE<br>b=-0.19<br>p=0.007	174<br>left ACC	$F_{(12,140)}$=2.89<br>p=0.001<br>R^2=0.21	thickxTAU<br>b=-0.2<br>p=0.007<br>depth<br>b=-0.13,<br>p=0.042<br>EDU<br>b=0.14,<br>p=0.033<br>AGE<br>b=-0.17,<br>p=0.024
179<br>left<br>DLPFC	$F_{(12,136)}$=3.8<br>p<0.0001<br>R^2=0.27	gyrixTAU<br>b=-0.18,<br>p=0.036<br>AGE<br>b=-0.35,<br>p<0.0001	178<br>left<br>DMPFC	$F_{(12,142)}$=2.83<br>p=0.002<br>R^2=0.21	depthxTAU<br>b=-0.16<br>p=0.037<br>EDU<br>b=0.16<br>p=0.03<br>AGE<br>b=-0.23<br>p=0.002	180<br>left<br>DLPFC	$F_{(12,139)}$=3.65<br>p=0.0001<br>R^2=0.26	depthxTAU<br>b=-0.19<br>p=0.004<br>gyri<br>b=0.17,<br>p=0.035<br>EDU<br>b=0.25<br>p=0.0001
189<br>left<br>DMPFC	$F_{(12,139)}$=5.2<br>p<0.0001<br>R^2=0.33	gyrixTAU<br>b=-0.17<br>p=0.028<br>AGE<br>b=-0.37<br>p<0.0001<br>gyrixEDU<br>b=0.28,<br>p=0.0001	180<br>left<br>DLPFC	$F_{(12,142)}$=2.14<br>p=0.018<br>R^2=0.16	depthxTAU<br>b=-0.16<br>p=0.035<br>EDU<br>b=0.22<br>p=0.003<br>AGE<br>b=-0.17<br>p=0.029	189<br>left<br>DMPFC	$F_{(12,139)}$=3.63<br>p=0.0001<br>R^2=0.26	gyrixTAU<br>b=-0.19<br>p=0.017<br>depthxTAU<br>b=-0.19<br>p=0.016<br>gyrixEDU<br>b=0.27<br>p=0.0002<br>AGE<br>b=-0.25<br>p=0.0006
197<br>left PCC	$F_{(12,138)}$=2.16<br>p=0.018<br>R^2=0.17	thickxTAU<br>b=-0.2,<br>p=0.037	189<br>left<br>DMPFC	$F_{(12,141)}$=4.13<br>p<0.0001<br>R^2=0.28	gyrixTAU<br>b=-0.22<br>p=0.007	191<br>left<br>precuneus	$F_{(12,138)}$=3.11<br>p=0.0007<br>R^2=0.23	thickxTAU<br>b=0.16<br>p=0.021

		AGE<br>b=-0.33,<br>p<0.0001			depthxTAU<br>b=-0.28<br>p=0.001<br>depthxEDU<br>b=0.17<br>p=0.013<br>gyrixEDU<br>b=0.29,<br>p=0.0001<br>AGE<br>b=-0.27<br>p=0.0002			depthxEDU<br>b=0.25<br>p=0.0006<br>gyrixEDU<br>b=0.19,<br>p=0.005<br>AGE<br>b=-0.25<br>p=0.0007
364<br>right<br>IPL	$F_{(12,137)}$=2.23<br>p=0.014<br>R^2=0.18	depthxTAU<br>b=-0.15<br>p=0.03<br>AGE<br>b=-0.29<br>p<0.0001	191<br>left<br>PCC/<br>precuneus	$F_{(12,141)}$=3.28<br>p=0.0003<br>R^2=0.23	thickxTAU<br>b=0.19<br>p=0.016<br>depthxEDU<br>b=0.21<br>p=0.007<br>gyrixEDU<br>b=0.23,<br>p=0.002<br>AGE<br>b=-0.29<br>p=0.0004	364<br>right<br>IPL	$F_{(12,141)}$=3.6<br>p=0.0001<br>R^2=0.25	depthxTAU<br>b=-0.19<br>p=0.003<br>depthxEDU<br>b=0.19<br>p=0.006<br>AGE<br>b=-0.21<br>p=0.002
366<br>right<br>IPL	$F_{(12,139)}$=3.6<br>p=0.0001<br>R^2=0.25	gyrixTAU<br>b=0.17<br>p=0.044<br>thickxTAU<br>b=0.18<br>p=0.031<br>AGE<br>b=-0.33<br>p<0.0001	199<br>left<br>precuneus	$F_{(12,141)}$=2.61<br>p=0.004<br>R^2=0.19	gyrixTAU<br>b=-0.19<br>p=0.028<br>gyrixEDU<br>b=0.23<br>p=0.007<br>AGE<br>b=-0.21<br>p=0.005	365<br>right<br>IPL	$F_{(12,139)}$=2.47<br>p=0.006<br>R^2=0.19	gyrixTAU<br>b=0.19<br>p=0.016<br>thickxTAU<br>b=0.22<br>p=0.007<br>EDU<br>b=0.23<br>p=0.001
367<br>right<br>MTG	$F_{(12,138)}$=2.79<br>p=0.002<br>R^2=0.21	gyrixTAU<br>b=-0.19,<br>p=0.019<br>depthxTAU<br>b=-0.19,<br>p=0.036<br>AGE<br>b=-0.29,<br>p<0.0001	364<br>right<br>IPL	$F_{(12,141)}$=3.49<br>p=0.0001<br>R^2=0.25	depthxTAU<br>b=-0.17<br>p=0.014<br>depthxEDU<br>b=0.2<br>p=0.005<br>AGE<br>b=-0.19<br>p=0.007	366<br>right<br>IPL	$F_{(12,141)}$=4<br>p<0.0001<br>R^2=0.27	gyrixTAU<br>b=0.22<br>p=0.007<br>thickxTAU<br>b=0.21<br>p=0.009<br>depthxTAU<br>b=-0.19<br>p=0.014<br>EDU<br>b=0.20<br>p=0.002<br>AGE<br>b=-0.18<br>p=0.011
369<br>right<br>STG	$F_{(12,137)}$=3.16<br>p=0.0006<br>R^2=0.23	gyrixTAU<br>b=0.26<br>p=0.0009<br>AGE<br>b=-0.31<br>p<0.0001	365<br>right<br>IPL	$F_{(12,141)}$=3.09<br>p=0.0007<br>R^2=0.22	depthxTAU<br>b=-0.16<br>p=0.04<br>thickxTAU<br>b=0.21,<br>p=0.019<br>EDU<br>b=0.19	367<br>right MTG	$F_{(12,139)}$=2.95<br>p=0.001<br>R^2=0.22	depthxTAU<br>b=-0.18,<br>p=0.034<br>gyrixTAU<br>b=-0.17,<br>p=0.029<br>gyrixEDU<br>b=0.14,

					p=0.008<br>AGE<br>b=-0.19<br>p=0.011			p=0.031<br>AGE<br>b=-0.17,<br>p=0.021
370<br>right<br>STG	$F_{(12,138)}$=2.32<br>p=0.01<br>R^2=0.18	depthxTAU<br>b=0.25<br>p=0.018<br>AGE<br>b=-0.34<br>p<0.0001	366<br>right<br>IPL	$F_{(12,142)}$=4.22<br>p<0.0001<br>R^2=0.28	gyrixTAU<br>b=0.23<br>p=0.007<br>thickxTAU<br>b=0.19<br>p=0.023<br>gyrixEDU<br>b=-0.17<br>p=0.024<br>AGE<br>b=-0.21<br>p=0.004	369<br>right<br>STG	$F_{(12,141)}$=2.09<br>p=0.022<br>R^2=0.16	gyrixTAU<br>b=0.20<br>p=0.014<br>EDU<br>b=0.21<br>p=0.004<br>AGE<br>b=-0.20<br>p=0.005
371<br>right<br>MTG	$F_{(12,140)}$=3.44<br>p=0.0002<br>R^2=0.24	depthxTAU<br>b=-0.41<br>p=0.0001<br>depthxEDU<br>b=0.21<br>p=0.017<br>AGE<br>b=-0.38<br>p<0.0001	367<br>right<br>MTG	$F_{(12,141)}$=2.94<br>p=0.001<br>R^2=0.21	depthxTAU<br>b=-0.22<br>p=0.014<br>EDU<br>b=0.19<br>p=0.011<br>AGE<br>b=-0.22<br>p=0.004	371<br>right<br>MTG	$F_{(12,139)}$=4.01<br>p<0.0001<br>R^2=0.27	depthxTAU<br>b=-0.42<br>p<0.0001<br>depthxEDU<br>b=0.18<br>p=0.023<br>gyri<br>b=0.13,<br>p=0.026<br>AGE<br>b=-0.22<br>p=0.002
374<br>right<br>MTG	$F_{(12,139)}$=3.23<br>p=0.0004<br>R^2=0.24	gyrixTAU<br>b=-0.20<br>p=0.013<br>AGE<br>b=-0.35<br>p<0.0001	369<br>right<br>STG	$F_{(12,142)}$=2.25<br>p=0.012<br>R^2=0.17	gyrixTAU<br>b=0.18<br>p=0.041<br>EDU<br>b=0.22<br>p=0.004<br>AGE<br>b=-0.24<br>p=0.001	373<br>right<br>MTG	$F_{(12,139)}$=2.43<br>p=0.007<br>R^2=0.19	depthxTAU<br>b=-0.33<br>p=0.003<br>EDU<br>b=0.22<br>p=0.002<br>AGE<br>b=-0.23<br>p=0.002
382<br>right<br>DMPFC	$F_{(12,136)}$=5.41<br>p<0.0001<br>R^2=0.35	depthxTAU<br>b=-0.20<br>p=0.008<br>depthxEDU<br>b=0.19,<br>p=0.005<br>thick<br>b=0.19,<br>p=0.006<br>AGE<br>b=-0.32<br>p<0.0001	371<br>right<br>MTG	$F_{(12,142)}$=2.71<br>p=0.002<br>R^2=0.2	depthxTAU<br>b=-0.29<br>p=0.006<br>depthxEDU<br>b=0.2<br>p=0.025<br>AGE<br>b=-0.25<br>p=0.002	376<br>right OFC	$F_{(12,140)}$=2.72<br>p=0.003<br>R^2=0.2	thickxTAU<br>b=0.17,<br>p=0.039<br>AGE<br>b=-0.19,<br>p=0.007
386<br>right<br>DLPFC	$F_{(12,138)}$=3.38<br>p=0.0002<br>R^2=0.24	depthxTAU<br>b=-0.25,<br>p=0.004<br>gyrixEDU<br>b=0.14,<br>p=0.025<br>AGE	373<br>right<br>MTG	$F_{(12,141)}$=1.89<br>p=0.001<br>R^2=0.15	depthxTAU<br>b=-0.26<br>p=0.038<br>EDU<br>b=0.20<br>p=0.01<br>AGE	378<br>right<br>DLPFC	$F_{(12,140)}$=2.57<br>p=0.004<br>R^2=0.19	gyrixTAU<br>b=-0.24<br>p=0.032<br>EDU<br>b=0.17<br>p=0.012<br>AGE

		b=-0.3,<br>p=0.0001			b=-0.26<br>p=0.001			b=-0.2<br>p=0.003
387<br>right<br>DMPFC	$F_{(12,137)}=2.97$<br>p=0.001<br>$R^2=0.22$	gyrixTAU<br>b=-0.19<br>p=0.012<br>AGE<br>b=-0.24<br>p=0.0008	376<br>right<br>OFC	$F_{(12,141)}=2.93$<br>p=0.001<br>$R^2=0.21$	thickxTAU<br>b=0.21<br>p=0.018<br>depthxTAU<br>b=-0.19,<br>p=0.04<br>gyrixEDU<br>b=0.19,<br>p=0.015<br>AGE<br>b=-0.21<br>p=0.005	382<br>right<br>DMPFC	$F_{(12,141)}=4.24$<br>p<0.0001<br>$R^2=0.28$	depthxTAU<br>b=-0.22<br>p=0.005<br>thick<br>b=0.31,<br>p<0.0001<br>gyrixEDU<br>b=0.18<br>p=0.025
388<br>right<br>DLPFC	$F_{(12,138)}=3.86$<br>p<0.0001<br>$R^2=0.27$	depthxTAU<br>b=-0.17<br>p=0.029<br>gyrixEDU<br>b=-0.14,<br>p=0.038<br>AGE<br>b=-0.37<br>p<0.0001	382<br>right<br>DMPFC	$F_{(12,141)}=3.54$<br>p=0.0001<br>$R^2=0.25$	depthxTAU<br>b=-0.17<br>p=0.043<br>thick<br>b=0.28,<br>p=0.0005<br>gyri<br>b=0.15,<br>p=0.035<br>EDU<br>b=0.21<br>p=0.004<br>AGE<br>b=-0.15<br>p=0.045	386<br>right<br>DLPFC	$F_{(12,139)}=3.22$<br>p=0.0005<br>$R^2=0.23$	depthxTAU<br>b=-0.19<br>p=0.02<br>depthxEDU<br>b=0.17<br>p=0.007<br>AGE<br>b=-0.17<br>p=0.021
390<br>right<br>DLPFC	$F_{(12,137)}=3.08$<br>p=0.0007<br>$R^2=0.23$	depthxTAU<br>b=-0.23<br>p=0.004<br>AGE<br>b=-0.26<br>p=0.0005	388<br>right<br>DLPFC	$F_{(12,142)}=3.51$<br>p=0.0001<br>$R^2=0.25$	depthxTAU<br>b=-0.26<br>p=0.002<br>EDU<br>b=0.16<br>p=0.024<br>AGE<br>b=-0.24<br>p=0.001	387<br>right<br>DMPFC	$F_{(12,139)}=2.57$<br>p=0.004<br>$R^2=0.2$	gyrixTAU<br>b=-0.18<br>p=0.021<br>EDU<br>b=0.018<br>p=0.009<br>AGE<br>b=-0.18<br>p=0.008
392<br>right<br>PCC	$F_{(12,139)}=3.07$<br>p=0.0008<br>$R^2=0.23$	gyrixTAU<br>b=-0.27<br>p=0.001<br>AGE<br>b=-0.39<br>p<0.0001	392<br>right<br>PCC	$F_{(12,142)}=3.2$<br>p=0.0005<br>$R^2=0.23$	gyrixTAU<br>b=-0.24<br>p=0.004<br>depthxEDU<br>b=0.20<br>p=0.01<br>thickxEDU<br>b=-0.19,<br>p=0.006<br>AGE<br>b=-0.19<br>p=0.013	388<br>right<br>DLPFC	$F_{(12,140)}=4.13$<br>p<0.0001<br>$R^2=0.28$	depthxTAU<br>b=-0.25<br>p=0.0009<br>gyrixEDU<br>b=0.22<br>p=0.0007<br>AGE<br>b=-0.22<br>p=0.002

			400 right precuneus	$F_{(12,142)}$=2.15 p=0.018 R^2=0.17	depthxTAU b=-0.21 p=0.008 EDU b=0.16 p=0.035 AGE b=-0.18 p=0.018	389 right DLPFC	$F_{(12,139)}$=2.4 p=0.008 R^2=0.18	depthxTAU b=-0.16 p=0.04 gyri b=0.15, p=0.023 EDU b=0.22 p=0.001 AGE b=-0.19 p=0.01
						391 right DLPFC	$F_{(12,139)}$=2.41 p=0.007 R^2=0.19	thickxTAU b=0.26 p=0.041 EDU b=0.2 p=0.003 AGE b=-0.21 p=0.004
						392 right PCC	$F_{(12,140)}$=2.54 p=0.005 R^2=0.19	gyrixTAU b=-0.21 p=0.008 thickxEDU b=-0.13 p=0.041 AGE b=-0.19 p=0.009

ACC= anterior cingulate cortex, DMPFC= dorsomedial prefrontal cortex, DLPFC= dorsolateral prefrontal cortex, IFC= inferior frontal cortex, IPL= inferior parietal lobule, MFC= middle frontal cortex, MTG= middle temporal gyrus, OFC= orbitofrontal cortex, PCC= posterior cingulate cortex, PO= pars orbitalis, STG= superior temporal gyrus, VMPFC= ventromedial prefrontal cortex.

Supplementary Table 3. Models' statistics of the significant association between DMN structural measures and Tau SUVRs in predicting Y2 encoding, retrieval and recall performance in A- individuals.

**Amyloid Negative -Y2**								
**Encoding-N**			**Retrieval- R**			**Recall- M**		
**ROI**	**Model**	**Predictors**	**ROI**	**Model**	**Predictors**	**ROI**	**Model**	**Predictors**
382 right DMPFC	$F_{(12,87)}$=4.61 p<0.0001 R^2=0.43	depthxTAU b=0.26 p=0.005 depthxEDU b=-0.48, p<0.0001	152 left MTG	$F_{(12,86)}$=3.34 p=0.0006 R^2=0.35	gyrixTAU b=-0.63 p=0.025 depthxEDU b=-0.32, p=0.001 thickxEDU b=0.16, p=0.02 gyrixEDU b=0.37,	149 left MTG	$F_{(12,89)}$=2.81 p=0.003 R^2=0.3	depthxTAU b=0.91 p=0.001 EDU b=-0.21 p=0.018

					p=0.001			
			175<br>left<br>PO	$F_{(12,89)}$=4.46<br>p<0.0001<br>R^2=0.41	gyrixTAU<br>b=0.27<br>p=0.01<br>gyrixEDU<br>b=-0.44,<br>p<0.0001	163<br>left<br>IPL	$F_{(12,89)}$=3.18<br>p=0.001<br>R^2=0.3	gyrixTAU<br>b=-0.47<br>p=0.0007<br>thickxTAU<br>b=0.32<br>p=0.03<br>EDU<br>b=-0.27<br>p=0.007
						174<br>left ACC	$F_{(12,86)}$=2.6<br>p=0.006<br>R^2=0.3	thickxTAU<br>b=0.25,<br>p=0.026<br>depthxTAU<br>b=0.29,<br>p=0.03
						185<br>left<br>DLPFC	$F_{(12,89)}$=3.27<br>p=0.0007<br>R^2=0.34	depthxTAU<br>b=-0.38<br>p=0.0001<br>thickxTAU<br>b=-0.19<br>p=0.009<br>depthxEDU<br>b=0.27,<br>p=0.016
						191<br>left<br>PCC/<br>precuneus	$F_{(12,91)}$=2.81<br>p=0.003<br>R^2=0.3	depthxTAU<br>b=0.29<br>p=0.017<br>depthxEDU<br>b=-0.42,<br>p=0.0004<br>gyrixEDU<br>b=-0.28,<br>p=0.003
						371<br>right<br>MTG	$F_{(12,88)}$=2.73<br>p=0.004<br>R^2=0.3	thickxTAU<br>b=-0.42<br>p=0.001<br>depthxTAU<br>b=0.34<br>p=0.021<br>thickxEDU<br>b=0.29<br>p=0.005
						372<br>right<br>MTG	$F_{(12,87)}$=2.74<br>p=0.004<br>R^2=0.31	thickxTAU<br>b=-0.41<br>p=0.024<br>depthxEDU<br>b=0.32<br>p=0.007<br>thickxEDU<br>b=0.35<br>p=0.003
						373<br>right MTG	$F_{(12,87)}$=2.6<br>p=0.006	thickxTAU<br>b=-0.3,

							R^2=0.30	p=0.028<br>thickxEDU<br>b=0.23,<br>p=0.034
						392<br>right PCC	$F_{(12,90)}$=2.98<br>p=0.002<br>R^2=0.32	gyrixTAU<br>b=0.27,<br>p=0.008<br>depthxTAU<br>b=-0.28,<br>p=0.013<br>EDU<br>b=-0.2,<br>p=0.036

DMPFC= dorsomedial prefrontal cortex, DLPFC= dorsolateral prefrontal cortex, IPL= inferior parietal lobule, MTG= middle temporal gyrus, PCC= posterior cingulate cortex, PO= pars orbitalis.

Supplementary Table 4. Models' statistics of the significant association between FPN structural measures and MMSE in predicting baseline encoding, retrieval and recall performance in A+ individuals.

**Amyloid Positive – Baseline**								
**Encoding- N**			**Retrieval- R**			**Recall- M**		
**ROI**	**Model**	**Predictors**	**ROI**	**Model**	**Predictors**	**ROI**	**Model**	**Predictors**
127	$F_{(16,123)}$=6.1<br>p<0.0001<br>R^2=0.48	thickxMMSE<br>b=0.26,<br>p=0.0005<br>gyrixMMSE<br>b=0.28,<br>p=0.011<br>depthxEDU<br>b=-0.21,<br>p=0.006<br>TAU<br>b=-0.31,<br>p=0.028<br>AGE<br>b=-0.28,<br>p=0.001	127	$F_{(16,122)}$=4.38<br>p<0.0001<br>R^2=0.4	thickxMMSE<br>b=0.16,<br>p=0.047<br>depthxMMSE<br>b=0.25,<br>p=0.017<br>AGE<br>b=-0.19<br>p=0.04<br>TAU<br>b=-0.42,<br>p=0.005	127	$F_{(16,123)}$=6.16<br>p<0.0001<br>R^2=0.48	thickxMMSE<br>b=0.29,<br>p=0.0002<br>gyrixMMSE<br>b=0.22,<br>p=0.043<br>AGE<br>b=-0.18,<br>p=0.031<br>TAU<br>b=-0.46,<br>p=0.001
128	$F_{(16,123)}$=4.21<br>p<0.0001<br>R^2=0.39	depthxMMSE<br>b=-0.19,<br>p=0.049<br>depthxTAU<br>b=-0.23,<br>p=0.035<br>AGE<br>b=-0.23,<br>p=0.015	134	$F_{(16,122)}$=5.35<br>p<0.0001<br>R^2=0.45	depthxMMSE<br>b=0.24,<br>p=0.02<br>depthxEDU<br>b=-0.22,<br>p=0.006<br>AGE<br>b=-0.19,<br>p=0.023<br>TAU<br>b=-0.22,<br>p=0.014	130	$F_{(16,121)}$=8.43<br>p<0.0001<br>R^2=0.56	thickxMMSE<br>b=0.26,<br>p=0.0005<br>depth<br>b=0.22,<br>p=0.003<br>TAU<br>b=-0.38,<br>p=0.0003
130	$F_{(16,123)}$=5.53<br>p<0.0001<br>R^2=0.45	thickxMMSE<br>b=0.27,<br>p=0.001<br>AGE<br>b=-0.28,<br>p=0.003<br>TAU	138	$F_{(16,122)}$=4,52<br>p<0.0001<br>R^2=0.41	depthxMMSE<br>b=0.15,<br>p=0.049<br>depthxEDU<br>b=-0.19,<br>p=0.021	132	$F_{(16,123)}$=5.94<br>p<0.0001<br>R^2=0.47	thickxMMSE<br>b=0.2,<br>p=0.01<br>depthxTAU<br>b=-0.21,<br>p=0.001

		b=-0.39<br>p=0.0009						
131	$F_{(16,123)}$=4.33<br>p<0.0001<br>R^2=0.39	depthxMMSE<br>b=-0.24,<br>p=0.021<br>AGE<br>b=-0.23,<br>p=0.012<br>TAU<br>b=-0.30<br>p=0.008	140	$F_{(16,120)}$=4.75<br>p<0.0001<br>R^2=0.42	thickxMMSE<br>b=0.15,<br>p=0.035<br>AGE<br>b=-0.17,<br>p=0.049	133	$F_{(16,121)}$=7.13<br>p<0.0001<br>R^2=0.52	thickxMMSE<br>b=0.19,<br>p=0.033<br>AGE<br>b=-0.16,<br>p=0.038<br>TAU<br>b=-0.4,<br>p<0.0001
132	$F_{(16,123)}$=5.92<br>p<0.0001<br>R^2=0.47	thickxMMSE<br>b=0.2<br>p=0.009<br>depthxMMSE<br>b=-0.19,<br>p=0.04<br>depthxTAU<br>b=-0.23,<br>p=0.007<br>AGE<br>b=-0.26<br>p=0.002	142	$F_{(16,120)}$=4.41<br>p<0.0001<br>R^2=0.41	thickxMMSE<br>b=0.18,<br>p=0.043	134	$F_{(16,122)}$=5.77<br>p<0.0001<br>R^2=0.47	thickxMMSE<br>b=0.18,<br>p=0.038<br>TAU<br>b=-0.37,<br>p<0.0001
133	$F_{(16,123)}$=4.4<br>p<0.0001<br>R^2=0.4	thickxMMSE<br>b=0.19,<br>p=0.049<br>AGE<br>b=-0.29,<br>p=0.001<br>TAU<br>b=-0.34,<br>p=0.0006	144	$F_{(16,122)}$=4.47<br>p<0.0001<br>R^2=0.4	thickxMMSE<br>b=0.28,<br>p=0.015	136	$F_{(16,123)}$=6.42<br>p<0.0001<br>R^2=0.49	thickxMMSE<br>b=0.23,<br>p=0.003<br>depthxEDU<br>b=-0.18,<br>p=0.028<br>AGE<br>b=-0.17,<br>p=0.042<br>TAU<br>b=-0.47,<br>p<0.0001
136	$F_{(16,123)}$=4.84<br>p<0.0001<br>R^2=0.42	thickxMMSE<br>b=0.21,<br>p=0.011<br>AGE<br>b=-0.27,<br>p=0.003<br>TAU<br>b=-0.4,<br>p=0.001	146	$F_{(16,122)}$=3.7<br>p<0.0001<br>R^2=0.36	thickxMMSE<br>b=0.34,<br>p=0.023	137	$F_{(16,123)}$=5.56<br>p<0.0001<br>R^2=0.45	thickxMMSE<br>b=0.3,<br>p=0.007<br>AGE<br>b=-0.19,<br>p=0.029
137	$F_{(16,123)}$=4.43<br>p<0.0001<br>R^2=0.4	thickxMMSE<br>b=0.24,<br>p=0.037<br>AGE<br>b=-0.27,<br>p=0.003	339	$F_{(16,122)}$=5.28<br>p<0.0001<br>R^2=0.45	depthxMMSE<br>b=-0.23,<br>p=0.032<br>EDU<br>b=0.23,<br>p=0.005<br>TAU<br>b=-0.19,<br>p=0.042	138	$F_{(16,123)}$=6.05<br>p<0.0001<br>R^2=0.48	thickxMMSE<br>b=0.18,<br>p=0.031<br>depthxEDU<br>b=-0.18,<br>p=0.02<br>TAU<br>b=-0.38,<br>p=0.002
140	$F_{(16,123)}$=5.29<br>p<0.0001<br>R^2=0.44	thickxMMSE<br>b=0.15,<br>p=0.046<br>AGE<br>b=-0.3,<br>p=0.0009<br>TAU<br>b=-0.41,<br>p=0.0003	344	$F_{(16,122)}$=4.22<br>p<0.0001<br>R^2=0.39	gyrixMMSE<br>b=0.23,<br>p=0.034	139	$F_{(16,121)}$=6.14<br>p<0.0001<br>R^2=0.48	thickxMMSE<br>b=0.19,<br>p=0.037

141	$F_{(16,123)}$=5.63<br>p<0.0001<br>R^2=0.46	gyrixMMSE<br>b=-0.27,<br>p=0.006<br>gyrixTAU<br>b=-0.26,<br>p=0.013<br>AGE<br>b=-0.28,<br>p=0.0007	350	$F_{(16,122)}$=4.43<br>p<0.0001<br>R^2=0.4	thickxMMSE<br>b=0.18,<br>p=0.041	140	$F_{(16,123)}$=6.34<br>p<0.0001<br>R^2=0.49	thickxMMSE<br>b=0.17,<br>p=0.015<br>depthxTAU<br>b=-0.37,<br>p=0.032<br>gyri<br>b=-0.17,<br>p=0.03<br>AGE<br>b=-0.19,<br>p=0.027
145	$F_{(16,123)}$=4.04<br>p<0.0001<br>R^2=0.38	depthxMMSE<br>b=-0.22,<br>p=0.018<br>depthxEDU<br>b=0.2,<br>p=0.043<br>AGE<br>b=-0.29,<br>p=0.001	354	$F_{(16,121)}$=4.41<br>p<0.0001<br>R^2=0.4	gyrixMMSE<br>b=0.29,<br>p=0.006<br>AGE<br>b=-0.21,<br>p=0.014	141	$F_{(16,123)}$=7.02<br>p<0.0001<br>R^2=0.51	thickxMMSE<br>b=0.24,<br>p=0.02<br>gyrixMMSE<br>b=-0.23,<br>p=0.012<br>gyrixTAU<br>b=-0.28,<br>p=0.006<br>AGE<br>b=-0.17,<br>p=0.028
146	$F_{(16,123)}$=5.29<br>p<0.0001<br>R^2=0.44	thickxMMSE<br>b=0.29,<br>p=0.036<br>gyrixEDU<br>b=0.24,<br>p=0.01<br>AGE<br>b=-0.33,<br>p=0.0002<br>TAU<br>b=-0.37,<br>p=0.001	356	$F_{(16,122)}$=5.25<br>p<0.0001<br>R^2=0.44	gyrixMMSE<br>b=0.21,<br>p=0.036<br>depthxEDU<br>b=0.18,<br>p=0.045<br>thick<br>b=0.17,<br>p=0.04<br>AGE<br>b=-0.21,<br>p=0.012	142	$F_{(16,123)}$=5.39<br>p<0.0001<br>R^2=0.45	thickxMMSE<br>b=0.25,<br>p=0.007<br>TAU<br>b=-0.42,<br>p=0.0009
148	$F_{(16,123)}$=4.46<br>p<0.0001<br>R^2=0.4	thickxMMSE<br>b=0.19,<br>p=0.046<br>AGE<br>b=-0.25,<br>p=0.004<br>TAU<br>b=-0.32,<br>p=0.0006	358	$F_{(16,122)}$=4.91<br>p<0.0001<br>R^2=0.43	depthxMMSE<br>b=-0.35,<br>p=0.007<br>depthxEDU<br>b=0.2,<br>p=0.038<br>gyri<br>b=0.19,<br>p=0.018<br>AGE<br>b=-0.22,<br>p=0.011	144	$F_{(16,122)}$=6.2<br>p<0.0001<br>R^2=0.48	thickxMMSE<br>b=0.27,<br>p=0.01<br>TAU<br>b=-0.33,<br>p=0.013
334	$F_{(16,123)}$=4.41<br>p<0.0001<br>R^2=0.4	thickxMMSE<br>b=0.2,<br>p=0.014<br>gyrixTAU<br>b=-0.29,<br>p=0.031<br>AGE<br>b=-0.25,<br>p=0.004	360	$F_{(16,122)}$=4.6<br>p<0.0001<br>R^2=0.41	thickxMMSE<br>b=0.25,<br>p=0.035<br>gyrixTAU<br>b=-0.23,<br>p=0.025<br>depthxTAU<br>b=-0.29,<br>p=0.009<br>EDU<br>b=0.2,<br>p=0.015	145	$F_{(16,123)}$=4.75<br>p<0.0001<br>R^2=0.42	depthxMMSE<br>b=-0.21,<br>p=0.023<br>AGE<br>b=-0.18,<br>p=0.04<br>TAU<br>b=-0.3,<br>p=0.04

					AGE<br>b=-0.17,<br>p=0.043			
335	$F_{(16,123)}$=5.07<br>p<0.0001<br>R^2=0.43	thickxMMSE<br>b=0.23,<br>p=0.022<br>depthxEDU<br>b=0.26,<br>p=0.008<br>thickxEDU<br>b=0.2,<br>p=0.03<br>gyrixTAU<br>b=-0.18,<br>p=0.039<br>thickxTAU<br>b=0.31,<br>p=0.008<br>AGE<br>b=-0.23,<br>p=0.005				146	$F_{(16,123)}$=5.75<br>p<0.0001<br>R^2=0.46	thickxMMSE<br>b=0.37,<br>p=0.007<br>TAU<br>b=-0.36,<br>p=0.002
337	$F_{(16,123)}$=3.95<br>p<0.0001<br>R^2=0.37	depthxMMSE<br>b=-0.22,<br>p=0.02<br>AGE<br>b=-0.26,<br>p=0.003<br>thick<br>b=0.22,<br>p=0.036				148	$F_{(16,123)}$=5.36<br>p<0.0001<br>R^2=0.45	thickxMMSE<br>b=0.24,<br>p=0.012<br>TAU<br>b=-0.32,<br>p=0.0004
340	$F_{(16,123)}$=4.64<br>p<0.0001<br>R^2=0.41	thickxMMSE<br>b=0.25,<br>p=0.004<br>gyrixMMSE<br>b=0.23,<br>p=0.004<br>gyrixTAU<br>b=0.22,<br>p=0.004<br>AGE<br>b=-0.2,<br>p=0.019				334	$F_{(16,122)}$=7.57<br>p<0.0001<br>R^2=0.54	thickxMMSE<br>b=0.24,<br>p=0.001<br>depthxEDU<br>b=-0.15,<br>p=0.044<br>gyrixTAU<br>b=-0.32,<br>p=0.008
341	$F_{(16,123)}$=3.66<br>p<0.0001<br>R^2=0.35	thickxMMSE<br>b=0.18<br>p=0.042<br>AGE<br>b=-0.26,<br>p=0.003<br>TAU<br>b=-0.24,<br>p=0.012				335	$F_{(16,123)}$=5.18<br>p<0.0001<br>R^2=0.44	thickxMMSE<br>b=0.21,<br>p=0.03<br>thickxTAU<br>b=0.25,<br>p=0.027
344	$F_{(16,123)}$=5.35<br>p<0.0001<br>R^2=0.45	gyrixMMSE<br>b=0.21,<br>p=0.037<br>gyrixTAU<br>b=0.28,<br>p=0.013<br>AGE<br>b=-0.19,<br>p=0.036				337	$F_{(16,123)}$=6<br>p<0.0001<br>R^2=0.48	depthxMMSE<br>b=-0.27,<br>p=0.003<br>TAU<br>b=-0.23,<br>p=0.031<br>gyri<br>b=0.2,<br>p=0.022
346	$F_{(16,123)}$=5.75<br>p<0.0001	thickxMMSE<br>b=0.3,				340	$F_{(16,123)}$=5.32<br>p<0.0001	thickxMMSE<br>b=0.26,

	R^2=0.46	p=0.001<br>AGE<br>b=-0.29,<br>p=0.0004<br>TAU<br>b=-0.24,<br>p=0.008					R^2=0.44	p=0.002<br>gyrixMMSE<br>b=0.19,<br>p=0.012<br>gyrixTAU<br>b=0.17,<br>p=0.02
350	$F_{(16,123)}$=3.95<br>p<0.0001<br>R^2=0.37	thickxMMSE<br>b=0.2,<br>p=0.027<br>AGE<br>b=-0.27,<br>p=0.003<br>TAU<br>b=-0.34,<br>p=0.005				341	$F_{(16,123)}$=4.33<br>p<0.0001<br>R^2=0.39	thickxMMSE<br>b=0.19,<br>p=0.032<br>TAU<br>b=-0.23,<br>p=0.012
355	$F_{(16,123)}$=3.9<br>p<0.0001<br>R^2=0.37	depthxMMSE<br>b=0.22,<br>p=0.02<br>AGE<br>b=-0.22,<br>p=0.015				342	$F_{(16,123)}$=5.69<br>p<0.0001<br>R^2=0.46	thickxMMSE<br>b=0.24,<br>p=0.041<br>gyrixEDU<br>b=0.18,<br>p=0.02<br>TAU<br>b=-0.31,<br>p=0.0008
357	$F_{(16,123)}$=4.51<br>p<0.0001<br>R^2=0.4	thickxMMSE<br>b=0.29,<br>p=0.023<br>gyrixMMSE<br>b=0.25,<br>p=0.03<br>depthxTAU<br>b=-0.51,<br>p=0.014<br>AGE<br>b=-0.24,<br>p=0.004				344	$F_{(16,123)}$=5.48<br>p<0.0001<br>R^2=0.45	thickxMMSE<br>b=0.31,<br>p=0.009<br>gyrixMMSE<br>b=0.27,<br>p=0.01<br>TAU<br>b=-0.28,<br>p=0.026
358	$F_{(16,123)}$=5.51<br>p<0.0001<br>R^2=0.45	thickxMMSE<br>b=0.35,<br>p=0.016<br>thickxTAU<br>b=0.26,<br>p=0.015<br>gyri<br>b=0.17,<br>p=0.03<br>AGE<br>b=-0.3,<br>p=0.002				346	$F_{(16,123)}$=8.26<br>p<0.0001<br>R^2=0.56	thickxMMSE<br>b=0.26,<br>p=0.001<br>gyrixTAU<br>b=-0.2,<br>p=0.025<br>depthxTAU<br>b=0.34<br>p=0.01<br>AGE<br>b=-0.15,<br>p=0.037
360	$F_{(16,123)}$=5.14<br>p<0.0001<br>R^2=0.44	thickxMMSE<br>b=0.27,<br>p=0.021<br>AGE<br>b=-0.27,<br>p=0.001<br>TAU<br>b=-0.26,<br>p=0.018				347	$F_{(16,123)}$=4.58<br>p<0.0001<br>R^2=0.41	thickxMMSE<br>b=-0.19,<br>p=0.027
						350	$F_{(16,123)}$=5.58<br>p<0.0001<br>R^2=0.46	thickxMMSE<br>b=0.24,<br>p=0.005<br>gyri

								b=-0.17,<br>p=0.03<br>TAU<br>b=-0.32,<br>p=0.004
						354	$F_{(16,123)}$=4.81<br>$p<0.0001$<br>R^2=0.42	gyrixMMSE<br>b=0.3,<br>p=0.005<br>gyrixTAU<br>b=0.25,<br>p=0.024
						357	$F_{(16,123)}$=4.27<br>$p<0.0001$<br>R^2=0.39	gyrixMMSE<br>b=0.23<br>p=0.049<br>depthxTAU<br>b=-0.48,<br>p=0.022
						358	$F_{(16,123)}$=6.55<br>$p<0.0001$<br>R^2=0.5	thickxMMSE<br>b=0.37,<br>p=0.007<br>thickxTAU<br>b=0.24,<br>p=0.02<br>gyri<br>b=0.2,<br>p=0.008<br>AGE<br>b=-0.2,<br>p=0.013
						360	$F_{(16,123)}$=5.34<br>$p<0.0001$<br>R^2=0.45	thickxMMSE<br>b=0.28,<br>p=0.016<br>depthxTAU<br>b=-0.23,<br>p=0.027
						361	$F_{(16,120)}$=8.47<br>$p<0.0001$<br>R^2=0.57	depthxMMSE<br>b=-0.24,<br>p=0.037<br>depthxEDU<br>b=0.23,<br>p=0.011<br>gyrixEDU<br>b=0.24,<br>p=0.003<br>thick<br>b=0.17,<br>p=0.025<br>TAU<br>b=-0.29,<br>p=0.005

Supplementary Table 5. Models' statistics of the significant association between FPN structural measures and Tau SUVRs in predicting baseline encoding, retrieval and recall performance in A- individuals.

**Amyloid Negative – Baseline**								
**Encoding- N**			**Retrieval- R**			**Recall- M**		
**ROI**	**Model**	**Predictors**	**ROI**	**Model**	**Predictors**	**ROI**	**Model**	**Predictors**

127	$F_{(12,138)}$=2.97<br>p=0.001<br>R^2=0.22	thickxTAU<br>b=0.3,<br>p=0.009<br>depthxTAU<br>b=0.25,<br>p=0.011<br>AGE<br>b=-0.33,<br>p<0.0001	127	$F_{(12,142)}$=2.75<br>p=0.002<br>R^2=0.2	thickxTAU<br>b=0.36,<br>p=0.004<br>thickxEDU<br>b=-0.19,<br>p=0.02<br>AGE<br>b=-0.18,<br>p=0.023	127	$F_{(12,139)}$=3.23<br>p=0.0004<br>R^2=0.23	thickxTAU<br>b=0.35,<br>p=0.001<br>thickxEDU<br>b=-0.14,<br>p=0.044<br>depthxTAU<br>b=0.2,<br>p=0.029<br>AGE<br>b=-0.15,<br>p=0.027
135	$F_{(12,139)}$=3.15<br>p=0.0006<br>R^2=0.23	depthxTAU<br>b=0.13,<br>p=0.04<br>AGE<br>b=-0.38,<br>p<0.0001	132	$F_{(12,141)}$=2.91<br>p=0.001<br>R^2=0.21	depthxTAU<br>b=-0.26,<br>p=0.027<br>depthxEDU<br>b=0.22,<br>p=0.005<br>AGE<br>b=-0.26,<br>p=0.001	339	$F_{(12,140)}$=2.79<br>p=0.002<br>R^2=0.21	thickxTAU<br>b=0.27,<br>p=0.018<br>depth<br>b=-0.17,<br>p=0.016<br>EDU<br>b=0.16,<br>p=0.015<br>AGE<br>b=-0.2,<br>p=0.013
339	$F_{(12,137)}$=2.18<br>p=0.016<br>R^2=0.17	thickxTAU<br>b=0.24,<br>p=0.032<br>AGE<br>b=-0.29,<br>p=0.0004	141	$F_{(12,141)}$=2.94<br>p=0.001<br>R^2=0.21	depthxTAU<br>b=-0.27,<br>p=0.013<br>thickxEDU<br>b=-0.18,<br>p=0.032<br>AGE<br>b=-0.26,<br>p=0.001	343	$F_{(12,139)}$=2.72<br>p=0.012<br>R^2=0.18	depthxTAU<br>b=-0.21,<br>p=0.013<br>EDU<br>b=0.18,<br>p=0.005
343	$F_{(12,137)}$=3<br>p=0.001<br>R^2=0.22	depthxTAU<br>b=-0.19,<br>p=0.023<br>thickxEDU<br>b=0.15,<br>p=0.048<br>gyrixEDU<br>b=0.15,<br>p=0.044<br>AGE<br>b=-0.25,<br>p=0.0006	336	$F_{(12,142)}$=2.11<br>p=0.02<br>R^2=0.16	thickxTAU<br>b=0.26,<br>p=0.017<br>AGE<br>b=-0.2,<br>p=0.011	348	$F_{(12,140)}$=3.06<br>p=0.0008<br>R^2=0.22	depthxTAU<br>b=-0.18,<br>p=0.005<br>thick<br>b=0.18,<br>p=0.015<br>EDU<br>b=0.17,<br>p=0.015
350	$F_{(12,139)}$=3.07<br>p=0.0008<br>R^2=0.22	gyrixTAU<br>b=0.17,<br>p=0.025	343	$F_{(12,142)}$=2.29<br>p=0.011<br>R^2=0.17	depthxTAU<br>b=-0.21,<br>p=0.029	350	$F_{(12,139)}$=3.65<br>p=0.0001<br>R^2=0.26	gyrixTAU<br>b=0.18,<br>p=0.008<br>gyrixEDU<br>b=-0.15,<br>p=0.023
			348	$F_{(12,142)}$=2.58<br>p=0.004<br>R^2=0.19	depthxTAU<br>b=-0.19,<br>p=0.009<br>AGE<br>b=-0.17,<br>p=0.034	354	$F_{(12,140)}$=2.51<br>p=0.005<br>R^2=0.19	depthxTAU<br>b=-0.19,<br>p=0.038<br>EDU<br>b=0.15,<br>p=0.032<br>AGE<br>b=-0.17,<br>p=0.017

Supplementary Table 6. Models' statistics of the significant association between FPN structural measures and Tau SUVRs in predicting Y2 encoding, retrieval and recall performance in A- individuals.

**Amyloid Negative – Y2**								
**Encoding- N**			**Retrieval- R**			**Recall- M**		
**ROI**	**Model**	**Predictors**	**ROI**	**Model**	**Predictors**	**ROI**	**Model**	**Predictors**
/	/	/	128	$F_{(12,86)}=2.81$<br>$p=0.003$<br>$R^2=0.31$	thickxTAU<br>b=-0.29,<br>p=0.019<br>depthxEDU<br>b=-0.15,<br>p=0.024<br>thickxEDU<br>b=0.28,<br>p=0.0002<br>gyri<br>b=0.17,<br>p=0.026	143	$F_{(12,89)}=4.05$<br>$p<0.0001$<br>$R^2=0.39$	gyrixTAU<br>b=0.3,<br>p=0.007<br>thickxEDU<br>b=0.27,<br>p=0.004<br>gyrixEDU<br>b=0.17,<br>p=0.049
			138	$F_{(12,88)}=3.6$<br>$p=0.0003$<br>$R^2=0.37$	thickxTAU<br>b=-0.38,<br>p<0.0001<br>gyrixTAU<br>b=-0.37,<br>p=0.0003<br>thickxEDU<br>b=0.21,<br>p=0.003<br>depthxTAU<br>b=-0.22,<br>p=0.017	342	$F_{(12,86)}=2.79$<br>$p=0.003$<br>$R^2=0.31$	depthxTAU<br>b=0.23,<br>p=0.035<br>gyrixEDU<br>b=0.32,<br>p=0.001<br>thick<br>b=-0.33,<br>p=0.003
			140	$F_{(12,88)}=3.52$<br>$p=0.0004$<br>$R^2=0.36$	gyrixTAU<br>b=0.27,<br>p=0.005<br>depthxEDU<br>b=0.22,<br>p=0.007<br>depthxTAU<br>b=-0.21,<br>p=0.02<br>thick<br>b=0.15,<br>p=0.022	350	$F_{(12,88)}=3.64$<br>$p=0.0002$<br>$R^2=0.37$	depthxTAU<br>b=0.21,<br>p=0.023<br>thickxTAU<br>b=-0.21,<br>p=0.014<br>thickxEDU<br>b=0.29,<br>p=0.002
			143	$F_{(12,89)}=4.47$<br>$p<0.0001$<br>$R^2=0.41$	gyrixTAU<br>b=0.34,<br>p=0.001	351	$F_{(12,86)}=3.53$<br>$p=0.0004$<br>$R^2=0.37$	thickxTAU<br>b=-0.2,<br>p=0.01<br>thickxEDU<br>b=0.36,<br>p<0.0001<br>depthxTAU<br>b=-0.25,<br>p=0.042
			346	$F_{(12,87)}=2.97$<br>$p=0.002$<br>$R^2=0.32$	thickxTAU<br>b=-0.34,<br>p=0.0009<br>gyrixEDU<br>b=-0.26,<br>p=0.0009			

References

1. Lemaitre, H., Goldman, A. L., Sambataro, F., Verchinski, B. A., Meyer-Lindenberg, A., Weinberger, D. R., & Mattay, V. S. (2012). Normal age-related brain morphometric changes: nonuniformity across cortical thickness, surface area and gray matter volume?. *Neurobiology of aging*, *33*(3), 617-e1.

2. Schwarz, C. G., Gunter, J. L., Wiste, H. J., Przybelski, S. A., Weigand, S. D., Ward, C. P., ... & Alzheimer's Disease Neuroimaging Initiative. (2016). A large-scale comparison of cortical thickness and volume methods for measuring Alzheimer's disease severity. *NeuroImage: Clinical*, *11*, 802-812.
